# Gene expression in TGFbeta-induced epithelial cell differentiation in a three-dimensional intestinal epithelial cell differentiation model

**DOI:** 10.1186/1471-2164-7-279

**Published:** 2006-10-31

**Authors:** Kati M Juuti-Uusitalo, Katri Kaukinen, Markku Mäki, Jarno Tuimala, Heikki Kainulainen

**Affiliations:** 1 Paediatric Research Centre, Tampere University and Tampere University Hospital, Tampere, Finland; 2 Department of Gastroenterology and Alimentary Tract Surgery, Tampere University Hospital, and University of Tampere, Medical School, Tampere, Finland; 3 Center for Scientific Computing, CSC, the Finnish IT Center for Science, Espoo, Finland; 4 Institute of Medical Technology, University of Tampere, Tampere, Finland; 5 Department of Biology of Physical Activity, University of Jyväskylä, Jyväskylä, Finland

## Abstract

**Background:**

The TGFβ1-induced signal transduction processes involved in growth and differentiation are only partly known. The three-dimensional epithelial differentiation model, in which T84 epithelial cells are induced to differentiate either with TGFβ1 or IMR-90 mesenchymal cell-secreted soluble factors, is previously shown to model epithelial cell differentiation seen in intestine. That model has not been used for large scale gene expression studies, such as microarray method. Therefore the gene expression changes were studied in undifferentiated and differentiated three-dimensional T84 cultures with cDNA microarray method in order to study the molecular changes and find new players in epithelial cell differentiation.

**Results:**

The expression of 372 genes out of 5188 arrayed sequences was significantly altered, and 47 of them were altered by both mediators. The data were validated and the altered genes are presented in ontology classes. For the genes tested the expressions in protein level were in accordance with the mRNA results. We also found 194 genes with no known function to be potentially important in epithelial cell differentiation. The mRNA expression changes induced by TGFβ1 were bigger than changes induced by soluble factors secreted by IMR-90 mesenchymal cells. The gene expression data was depicted in already known signaling pathway routes.

**Conclusion:**

Our results reveal potential new signaling pathways and several new genes affected by TGFβ in epithelial cell differentiation. The differentiation induced by TGFβ1 appears to be more potent than the differentiation induced by mesenchymal cells. This study indicates that our cell culture model is a suitable tool in studying regulatory mechanisms during epithelial cell differentiation in intestine. Furthermore the present results indicate that our model is a good tool for finding new players acting in the differentiation of epithelial cells.

## Background

The intestinal epithelium comprises multiple cell types which are progenitors of the stem cells located in crypt region. During their migration along the crypt axis epithelial cells differentiate from proliferative cells into secretory cells or absorptive enterocytes [1] Epithelial cell growth, motility and morphogenesis are controlled by soluble factors such as transforming growth factor beta (TGFβ) [1], a multifunctional growth factor [2-4]. Mesenchymal cells producing TGFβ are in turn regulated by various hormonal, paracrine and exogenous factors [5,6]. Epithelial cells can also regulate their own gene expression via negative regulatory feedback loops [7]. The role of the specific signaling pathways across the crypt axis, and the transcription factors controlling the crypt-specific expression of specific genes has been characterized [1], but the molecular events synergistically leading to the transition of crypt-like cells into absorptive enterocytes call for further study. The key to an understanding of cellular differentiation and development is elucidation of the molecular events regulating transcription. Research on epithelial cell differentiation in primary cell cultures has been hampered by the poor viability of stem cells [8], and the tumor cell lines capable of differentiating have thus been extensively used in studies of epithelial cell differentiation [8-11]. In a model created by our group, T84 epithelial cells grown three-dimensionally are induced to differentiate either by soluble factors secreted by mesenchymal cells (IMR-90 fibroblasts) or by addition of TGFβ1 [12,13]. T84 cells differentiated in this model are shown to express alkaline phosphatase and sialomucin, that are usually expressed by villus tip epithelial cells in normal duodenum, while the expression of c-met-proto oncogene has been shown to be down-regulated [12]. Differentiated T84 are shown to be negative for sulfomucin that is usually expressed in colon [12]. IMR fibroblast-induced differentiation of T84 cells has been shown to occur in a TGFβ1-dependent manner [12]. The advantage of our model is that differentiation commences only after induction, and the differentiation process can therefore be studied precisely. It has already been harnessed to study alterations in gene expression during epithelial cell differentiation by employing differential display [14-17]. By this method we have found several novel candidate genes, for example SAP30L, which might have a role in the epithelial cell differentiation [15].

TGFβ1-induced cell-type specific signal transduction networks warrants further clarification [2]. In the present study, using cDNA microarray and taking advantage of our three-dimensional epithelial cell culture, our aim was to find novel molecular changes in gene expression accompanying epithelial cell differentiation only in undifferentiated and differentiated cultures. Furthermore, we compared the gene expressions in epithelial cells mediated either by TGFβ1 or soluble factors excreted by IMR-90 mesenchymal cells.

## Results

In the three-dimensional epithelial cell differentiation model, crypt-like T84 epithelial cells were induced to differentiate either by TGFβ1 (hereafter called as TGFβ1-differentiated) or by soluble factors secreted by IMR-90 type human embryonic lung fibroblasts (hereafter called as IMR-differentiated). The T84 cells cultured within collagen gel supplemented with medium were used as undifferentiated controls [12,13]. The study focused on detecting changes in gene expression upon differentiation; we therefore studied undifferentiated and differentiated epithelial cells harvested after seven days of culturing. Altogether the transcription of 372 out of 5188 genes was found to be significantly altered when TGFβ1-differentiated and IMR-differentiated cultures were compared to the undifferentiated control culture. All the affected genes were grouped into ontology classes according to their known or predicted functions. From all significantly altered genes 8.6% attended on metabolism, other than energy metabolism (GO:0044237), and 90% of them were down-regulated. Genes in the subgroup of metabolism, the generation of precursor metabolites and energy (GO:0006091), were affected in 2% of all genes. 5.6% of genes controlled the cell cycle and DNA processing (nucleic acid binding (GO:0003676)), and 91% of them were down-regulated in differentiated cells. 17.5% of affected genes regulated transcription regulator activity (GO:0030528), and 83% of them were down-regulated in differentiated cells. Genes coding proteins for signal transducer activity (GO:0004871, GO:0007165, GO:0007154) was the largest functional group, 27% of all significantly altered genes. 13% of the genes that had significantly altered expression affected to protein folding i.e. cellular macromolecule metabolism (GO:0044260). Genes having a role to cell rescue and defense i.e. inflammatory response (GO:0006954) were 5% of all affected genes. All genes belonging to this group were down-regulated in differentiated cells. Equal amount of affected genes (6%) controlled cellular organization i.e. organelle organization and biogenesis (GO:0006996I) and transport (GO:0006810). One quarter (25%) of all affected genes had yet no known role in biological processes (GO:0000004). In IMR-differentiated cells several of the genes were slightly but insignificantly affected (the expression ratios were between 0.8 and 1.25). A full list of the genes in question is presented as an additional file (see Additional file 5).

### Gene ontology classes

We focused on genes which have been shown to function in transcription regulation, or signal transduction, or whose function remains unknown but putatively important in epithelial cell differentiation. The list of selected genes is presented in Table 1. Six of the affected genes are involved in the biosynthesis of cholesterol, and all of them were down-regulated (Table 1, ontology class A). Also genes associated with oxidative phosphorylation were decreased (Table 1, B), as well as those controlling the cell cycle and DNA processing (Table 1, C). CDK6 was the only selected gene in this class up-regulated in TGFβ1-differentiated but down-regulated in IMR-differentiated epithelial cells. In the group of genes controlling transcription and translation there were five zinc finger genes, all down-regulated (Table 1, D). The genes with a role in TGFβ-, the tyrosine kinase- and the wnt-signaling pathways, listed in the ontology class E, were all down-regulated, as were those regulating protein folding, for example cathepsin S and transglutaminase 2 (Table 1, ontology class F). Genes which evinced significantly altered expression and affected to cell rescue and defense (G), cellular organization (H) and transport (I) are presented only in the supplementary data. Of the 372 genes whose expression was changed, 94 (25%) had no function known so far. Some unknown genes potentially important in epithelial cell differentiation are listed in Table 1 as ontology class J.

**Table 1:** List of affected genes that had a significant change in their mRNA expression when TGFβ-differentiated T84 cells were compared to T84 cells grown solely in collagen I gel (= TGFβ-treated vs. control) and T84 cells differentiated by soluble factors secreted by mesenchymal cells were compared to T84 cells grown solely in collagen I gel (= IMR treated vs. control).

**Gene ontology class**	**Gene name**	**GenBank no**	**chromosomal location**	**TGFβ-treated vs. control**				**IMR-treated vs. control**			
				
				ratio	SD	p-value	FDR	ratio	SD	p-value	FDR
**A**	**DHCR24: 24-dehydrocholesterol reductase**	AA482324	**1p33-p31.1**	**0.68**↓	(±0.20)	0.699	0.733	1.01	(±0.14)	0.969	1.000
**A**	**FDPS: farnesyl diphosphate synthase (farnesyl pyrophosphate synthetase, dimethylallyltranstransferase, geranyltranstransferase)**	T66907 T65907	**1q22**	**0.29↓** **0.57↓**	(±0.24) (±0.45)	0.180 0.148	0.291 0.272	1.43 1.06	(±1.32) (±1.02)	0.807 0.499	1.000 1.000
**A**	**CYP1B1: cytochrome P450, family 1, subfamily B, polypeptide 1**	AA448157	**2p21**	**0.66**↓	(±0.26)	0.724	0.795	**0.68**↓	(±0.23)	0.716	1.000
**A**	**CYP3A4: cytochrome P450, subfamily IIIA (niphedipine oxidase), polypeptide 4**	R91078	**7q21.1**	**0.70**↓	(±0.05)	0.372	0.479	0.88	(±0.18)	0.520	1.000
**A**	**CYP2E: cytochrome P450, subfamily IIE (ethanol-inducible)**	H50500	**10q24.3-qter**	**0.58**↓	(±0.31)	0.509	0.607	0.96	(±0.60)	0.991	1.000
**A**	**CYP19A1: cytochrome P450, family 19, subfamily A, polypeptide 1**	R32428	**15q21.1**	**0.75**↓	(±0.08)	0.744	0.812	1.09	(±0.14)	0.923	1.000
**B**	**SDHB: succinate dehydrogenase complex, subunit B, iron sulfur (Ip)**	AA463565	**1p36.1-p35**	**0.43**↓	(±0.20)	0.041	0.265	0.81	(±0.38)	0.570	1.000
**B**	**UQCR: ubiquinol-cytochrome c reductase (6.4 kD) subunit**	R46837	**19p13.3**	**0.42**↓	(±0.40	0.123	0.265	0.90	(±0.49)	0.629	1.000
**C**	**CDK6: cyclin-dependent kinase 6**	H73724	**7q21-q22**	**2.57**↑	(±1.34)	0.418	0.525	**0.61**↓	(±0.34)	0.442	1.000
**D**	**EIF4E2: eukaryotic translation initiation factor 4E member 2**	W01534	**2q37**	**3.54**↑	(±1.90)	0.390	0.495	0.90	(±0.71)	0.499	1.000
**D**	**TFDP2: transcription factor Dp-2**	AA465444	**3q23**	**0.57**↓	(±0.18)	0.036	0.265	0.97	(±0.33)	0.920	1.000
**D**	**HNRPH1: heterogeneous nuclear ribonucleoprotein H1**	W96114	**5q35.3**	**0.53**↓	(±0.24)	0.572	0.665	0.98	(±0.39)	0.739	1.000
**D**	**ZNF193: zinc finger protein 193**	AA252169	**6p21.3**	0.49	(±0.20)	0.102	0.265	0.96	(±0.68)	0.482	1.000
**D**	**MYC: v-myc myelocytomatosis viral oncogene homolog (avian)**	AA464600	**8q24.12-q24.13**	0.78	(±0.42)	0.156	0.275	0.91	(±0.27)	0.800	1.000
**D**	**PRP18: pre-mRNA processing factor 18**	H82325	**10p12.33**	**0.52**↓	(±0.28)	0.086	0.265	0.97	(±0.22)	0.791	1.000
**D**	**TFDP1: transcription factor Dp-1**	W33012	**13q34**	**0.76**↓	(±0.009)	0.858	0.898	0.99	(±0.26)	0.957	1.000
**D**	**EIF2B2: eukaryotic translation initiation factor 2B, subunit 2 (beta, 39 kD)**	R86304	**14q24.3**	**0.64**↓	(±0.19)	0.018	0.261	1.05	(±0.25)	0.765	1.000
**D**	**ZNF161: zinc finger protein 161**	AA232647	**17q23.3**	**0.71**↓ **0.75**↓	(±0.11) (±0.13)	0.339	0.445	0.94 1.08	(±0.31) (±0.11)	0.608	1.000 1.000
**D**	**ZNF24: zinc finger protein 24 (KOX 17)**	AA447098	**18q12**	**0.73**↓	(±0.07)	0.651	0.732	1.10	(±0.46)	0.712	1.000
**D**	**ZNF358: Zinc finger protein 358**	H20045	**19p13**	**0.64**↓	(±0.09)	0.304	0.411	0.93	(±0.30)	0.621	1.000
**D**	**MRPL4: mitochondrial ribosomal protein L4**	AA490981	**19p13.2**	**0.66**↓	(±0.13)	0.030	0.265	0.90	(±0.16)	0.503	1.000
**D**	**RPS5: ribosomal protein S5**	AA456616	**19q13.4**	**0.66**↓	(±0.13)	0.030	0.265	0.90	(±0.16)	0.503	1.000
**D**	**EZF-2: endothelial zinc finger protein 2**	R63318	**19q13.43**	**0.58**↓	(±0.22)	0.061	0.265	0.87	(±0.23)	0.374	1.000
**D**	**ID1: inhibitor of DNA binding 1, dominant negative helix-loop-helix protein**	AA457158	**20q11**	**0.74**↓	(±0.18)	0.210	0.317	1.06	(±0.13)	0.807	1.000
**D**	**SF3A1 splicing factor 3a, subunit 1. 120 kD**	T72698	**22q12.2**	**0.71**↓	(±0.13)	0.510	0.609	1.11	(±0.36)	0.967	1.000
**E**	**CTNNBIP1: catenin, beta interacting protein 1**	R78539	**1p36.22**	**0.59**↓	(±0.33)	0.205	0.313	1.23	(±0.91)	0.836	1.000
**E**	**CTNNB1: catenin (cadherin-associated protein), beta 1, 88 kDa**	AA442092	**3p21**	**0.65**↓	(±0.06)	0.628	0.712	0.99	(±0.08)	0.948	1.000
**E**	**WNT5A: wingless-type MMTV integration site family, member 5A**	W49672	**3p21-p14**	**0.72**↓	(±0.19)	0.307	0.414	1.07	(±0.16)	0.899	1.000
**E**	**TGFBR2: transforming growth factor, beta receptor II (70–80 kD)**	AA487034	**3p22**	**0.71**↓	(±0.17)	0.450	0.555	1.06	(±0.14)	0.906	1.000
**E**	**SIAH2: seven in absentia homolog 2 (Drosophila)**	AA029041	**3q25**	**0.65**↓	(±0.13)	0.037	0.265	1.01	(±0.19)	0.948	1.000
**E**	**PIK3R1: phosphoinositide-3-kinase, regulatory subunit, polypeptide 1 (p85 alpha)**	R54050	**5q12-q13**	**0.61**↓	(±0.11)	0.539	0.636	1.25	(±0.75)	0.832	1.000
**E**	**PDGFRB: platelet-derived growth factor receptor, beta polypeptide**	R56211	**5q31-32**	1.01	(±0.29)	0.957	0.973	**1.23**↑	(±0.16)	0.390	1.000
**E**	**EGFR: epidermal growth factor receptor (erythroblastic leukemia viral (v-erb-b) oncogene homolog, avian)**	R35665 W48713	**7p12**	**0.47**↓ **0.75**↓	(±0.04) (±0.13)	0.022 0.444	0.265 0.550	1.02 1.09	(±0.63) (±0.10)	0.806 0.693	1.000 1.000
**E**	**IGF2: insulin-like growth factor 2 (somatomedin A)**	N54596	**11p15.5**	**0.57**↓ **0.63**↓	(±0.16) (±0.17)	0.241 0.335	0.345 0.441	0.92 1.23	(±0.27) (±0.86)	0.565 0.912	1.000 1.000
**E**	**SIAH1: seven in absentia homolog 1 (Drosophila)**	AA447531	**16q12**	**0.57**↓	(±0.23)	0.055	0.265	0.97	(±0.55)	0.920	1.000
**E**	**NLK: nemo-like kinase**	R70769	**17q11.2**	**0.55**↓	(±0.24)	0.298	0.504	0.85	(±0.39)	0.499	1.000
**E**	**SMAD4: SMAD, mothers against DPP homolog 4 (Drosophila)**	AA456439	**18q21.1**	**0.78**↓	(±0.14)	0.186	0.296	1.00	(±0.23)	0.870	1.000
**F**	**CTSS: cathepsin S**	AA236164	**1q21**	**0.53**↓	(±0.04)	0.327	0.433	0.90	(±0.30)	0.580	1.000
**F**	**PSMA3: proteasome (prosome, macropain) subunit, alpha type, 3**	AA465593	**14q23**	**0.58**↓	(±0.19	0.319	0.426	1.11	(±0.68)	0.700	1.000
**F**	**PSMB6: proteasome (prosome, macropain) subunit, beta type, 6**	AA070997	**17p13**	**0.76**↓	(±0.16)	0.093	0.265	1.00	(±0.12)	0.972	1.000
**F**	**TGM2: transglutaminase 2 (C polypeptide, protein-glutamine-gamma-glutamyltransferase)**	R97066	**20q12**	**0.62**↓	(±0.30)	0.171	0.284	0.73	(±0.24)	0.182	1.000
**H**	**KRT19:keratin 19**	AA464250	**17q21.2**	13.90	(±16.73)	0.390	0.496	19.95	(±12.99)	0.499	1.000
**J**	**LOC440582: similar to Peptidyl-prolyl cis-trans isomerase E (PPIase E) (Rotamase E) (Cyclophilin E) (Cyclophilin 33)**	W17246	**1p34.3**	**0.79**↓	(±0.20)	0.290	0.397	1.11	(±0.14)	0.293	1.000
**J**	**RP4-622L5: hypothetical protein RP4-622L5**	T85191	**1p36.11-p34.2**	**0.69**↓	(±0.13)	0.066	0.265	1.11	(±0.37)	0.673	1.000
**J**	**EST**	T99671	**2q36**	**0.66**↓	(±0.18)	0.610	0.698	0.99	(±0.30)	0.770	1.000
**J**	**FLJ12057**	N77990	**3q21**	**0.71**↓	(±0.10)	0.394	0.501	1.01	(±0.26)	0.840	1.000
**J**	**MGC2198**	H81199	**3q27-q28**	**0.57**↓	(±0.25)	0.258	0.364	1.21	(±0.97)	0.727	1.000
**J**	**EST**	R74480	**5**	**0.65**↓	(±0.09)	0.148	0.272	0.93	(±0.55)	0.458	1.000
**J**	**CCL28: chemokine (C-C motif) ligand 28**	R38459	**5p12**	**0.63**↓	(±0.13)	0.288	0.395	1.06	(±0.41)	0.905	1.000
**J**	**FCHO2: FCH domain only 2**	H93842	**5q13**	1.03	(±0.54)	0.859	0.900	**1.48**↑	(±0.44)	0.277	1.000
**J**	**Hypothetical gene supported by AK126569**	N92035	**5q23**	**0.52**↓	(±0.20)	0.161	0.277	0.94	(±0.31)	0.577	1.000
**J**	**SMG1: PI-3-kinase-related kinase SMG-1**	W32907	**6p12.3**	**0.70**↓	(±0.22)	0.207	0.315	1.36	(±0.55)	0.259	1.000
**J**	**cDNA DKFZp667D095**	R26163	**8p22**	**0.61**↓	(±0.32)	0.118	0.265	1.10	(±0.59)	0.857	1.000
**J**	**DPYS: hypothetical protein PRO2949**	N73761	**8q22**	**3.29↓**	(±1.73)	0.513	0.611	0.85	(±0.54)	0.566	1.000
**J**	**D12S2489E**	AA397819	**12p13.2-p12.3**	**0.66**↓	(±0.06)	0.011	0.227	0.92	(±0.19)	0.678	1.000
**J**	**ZNF629: Zinc finger protein 629**	AA128587	**16p11.1**	**0.55**↓	(±0.15)	0.157	0.275	0.93	(±0.33)	0.533	1.000
**J**	**cDNA FLJ32121 fis, clone PEBLM1000083**	T69477	**16q22**	**0.57**↓	(±0.18)	0.124	0.265	0.85	(±0.25)	0.698	1.000
**J**	**SFRS14: splicing factor, arginine/serine-rich 14**	AA485539	**19p12**	**0.70**↓	(±0.17)	0.201	0.310	1.88	(±1.38)	0.995	1.000
**J**	**PNPLA4: patatin-like phospholipase domain containing 4**	AA449678	**Xp22.3**	**0.58**↓	(±0.09)	0.387	0.495	0.94	(±0.33)	0.634	1.000
**J**	**LOC286467: hypothetical protein LOC286467**	R95805	**Xq26.1**	**2.85↑**	(±1.02)	0.293	0.400	**0.85**↓	(±0.42)	0.530	1.000

Genes are sorted by functional classification. A: Cellular metabolism (GO:0044237), Metabolism (other than energy metabolism), B: Generation of precursor metabolites and energy GO:0006091), C: Nucleic acid binding (GO:0003676), D: Transcription regulator activity (GO:0030528), E: Signal transducer activity (GO:0004871), Signal transduction (GO:0007165) and Cell communication (GO:0007154), F: Cellular macromolecule metabolism (GO:0044260), H: Organelle organization and biogenesis (GO:0006996I), I: Transport (GO:0006810), J: Biological process unknown (GO:0000004), Upward arrow denotes up-regulated mRNA expression, downward arrow down-regulated mRNA expression, bold indicates genes that have significant alteration in gene expression. Ratio is mean value calculated from three separate microarray experiments; SD is the standard deviation between separate experiments. Statistic probability, p-value, was calculated by using t-test. FDR is the calculated Benjamin-Hochberg false discovery rate.

Forty-seven out of the 372 affected genes were altered in both TGFβ1- and IMR-differentiated epithelial cells as compared to controls (Table 2); in 39 (85%) of them the expression changes were to same direction. Differentiation altered the expression of genes in all ontology groups. Altogether 14 affected genes had a role in cell communication and signal transduction (Table 2, ontology class E) and six genes affected in both sample groups had as yet no known function (Table 2, J).

**Table 2:** List of the 46 genes evincing a significant change in their mRNA expression both in TGFβ-treated compared to cultures grown in collagen I gel and medium (= TGFβ-treated vs. control) and IMR fibroblast soluble factor-differentiated cultures compared to cultures grown in collagen I gel and medium (= IMR-treated vs. control).

**Gene ontology class**	**Gene name**	**GenBank no**	**chromosomal location**	**TGFβ-treated vs. control**				**IMR-treated vs. control**				**ANOVA**
				
				ratio	SD	p-value	FDR	ratio	SD	p-value	FDR	p-value
**A**	**CYP1B1:cytochrome P450, family 1, subfamily B, polypeptide 1**	AA448157	**2p21**	**0.66**↓	(±0.26)	0.724	0.795	**0.68**↓	(±0.23)	0.716	1.000	0.669
**A**	**UMPS: uridine monophosphate synthetase (orotate phosphoribosyl transferase and orotidine-5'-decarboxylase)**	AA426227	**3q13**	**0.65**↓	(±0.56)	0.365	0.464	**0.68**↓	(±0.45)	0.270	1.000	0.173
**A**	**GM2A: GM2 ganglioside activator protein**	AA453978	**5q31.3-q33.1**	**0.36**↓	(±0.26)	0.041	0.265	**0.45**↓	(±0.47)	0.292	1.000	0.279
**A**	**AMD1: S-adenosylmethionine decarboxylase 1**	R82299	**6q21-q22**	**0.59**↓	(±0.28)	0.054	0.265	**0.80**↓	(±0.50)	0.410	1.000	0.420
**A**	**AHCY: S-adenosylhomocysteine hydrolase**	AA485626	**20cen-q13.1**	**0.65**↓	(±0.23)	0.081	0.265	**1.30**↑	(±0.42)	0.210	1.000	0.347
**A**	**HMOX1: heme oxygenase (decycling) 1**	T71757	**22q12**	**3.75**↑	(±2.58)	0.693	0.769	**0.70**↓	(±0.54)	0.491	1.000	0.456
**A**	**ALAS2: aminolevulinate, delta-, synthase 2 (sideroblastic/hypochromic anemia)**	AA410346	**Xp11.21**	**0.48**↓	(±0.15)	0.010	0.225	**0.80**↓	(±0.41)	0.299	1.000	0.322
**C**	**HIST1H2AC: histone 1, H2ac**	AA453105	**6p21.3**	**0.78**↓	(±0.24)	0.369	0.477	**0.78**↓	(±0.28)	0.180	1.000	0.196
**C**	**CDK6: cyclin-dependent kinase 6**	H73724	**7q21-q22**	**2.57**↑	(±1.34)	0.418	0.525	**0.61**↓	(±0.34)	0.442	1.000	0.524
**C**	**APC7: anaphase-promoting complex subunit 7**	T67474	**12q13.12**	**0.77**↓	(±0.07)	0.141	0.269	**0.80**↓	(±0.13)	0.221	1.000	0.211
**C**	**LASS4: LAG1 longevity assurance homolog 4 (S. cerevisiae)**	AA025779	**19p13.2**	**0.62**↓	(±0.29)	0.282	0.388	**0.73**↓	(±0.33)	0.339	1.000	0.268
**D**	**RLF: Rearranged L-myc fusion sequence**	R26070	**1p32**	**0.54**↓	(±0.27)	0.467	0.571	**0.77**↓	(±0.29)	0.540	1.000	0.437
**D**	**TCF7: transcription factor 7 (T-cell specific, HMG-box)**	AA480071	**5q31.1**	**0.76**↓	(±0.49)	0.180	0.291	**0.80**↓	(±0.42)	0.297	1.000	0.258
**D**	**TCF8: transcription factor 8 (represses interleukin 2 expression)**	R22087	**10p11.2**	**0.62**↓	(±0.38)	0.371	0.479	**0.74**↓	(±0.39)	0.392	1.000	0.286
**D**	**TCEA2: transcription elongation factor A (SII), 2**	AA412500	**20q13.33**	**0.61**↓	(±0.36)	0.241	0.345	**0.72**↓	(±0.33)	0.161	1.000	0.157
**E**	**TIE1: tyrosine kinase with immunoglobulin-like and EGF-like domains 1**	AA432062	**1p34-p33**	**0.68**↓	(±0.20)	0.084	0.265	**0.72**↓	(±0.13)	0.070	1.000	0.077
**E**	**GPA33: glycoprotein A33 (transmembrane)**	AA055862	**1q24.1**	**0.72**↓	(±0.10)	0.679	0.756	**0.80**↓	(±0.16)	0.752	1.000	0.703
**E**	**MAPKAPK2: mitogen-activated protein kinase-activated protein kinase 2**	AA455056	**1q32**	**0.61**↓	(±0.10)	0.031	0.265	**0.80**↓	(±0.21)	0.385	1.000	0.488
**E**	**PDZD2: PDZ domain containing 2**	AA405458	**5p13.2**	**0.62**↓	(±0.07)	0.049	0.265	**0.83**↓	(±0.12)	0.350	1.000	0.428
**E**	**PRL:prolactin**	AA133920	**6p22.2-p21.3**	**0.66**↓	(±0.31)	0.368	0.470	**0.69**↓	(±0.29)	0.375	1.000	0.357
**E**	**AKAP12:A kinase (PRKA) anchor protein (gravin) 12**	AA478542	**6q24-q25**	**0.75**↓	(±0.41)	0.246	0.351	**0.77**↓	(±0.35)	0.256	1.000	0.287
**E**	**STAM: signal transducing adaptor molecule (SH3 domain and ITAM motif) 1**	AA485996	**10p14-p13**	**0.56**↓	(±0.26)	0.186	0.297	**0.82**↓	(±0.16)	0.382	1.000	0.396
**E**	**CARP: cardiac ankyrin repeat protein, cytokine inducible nuclear protein C193**	AA488072	**10q23.31**	**0.77**↓	(±0.16)	0.038	0.265	**0.82**↓	(±0.17)	0.073	1.000	0.080
**E**	**MS4A1: membrane-spanning 4-domains, subfamily A, member 1 (Fc fragment of IgE, high affinity I, receptor for; beta polypeptide**	N91385	**11q12-q13.1**	**1.24**↑	(±0.66)	0.612	0.699	**1.63**↑	(±0.62)	0.323	1.000	0.302
**E**	**DRPLA: dentatorubral-pallidoluysian atrophy (atrophin-1)**	H08642	**12p13.31**	**0.65**↓	(±0.42)	0.330	0.436	**0.80**↓	(±0.53)	0.364	1.000	0.256
**E**	**PRKCB1: protein kinase C, beta 1/cDNA DKFZp761J0720**	AA479102	**16p11.2**	**1.70**↑	(±0.75)	0.392	0.499	**0.69**↓	(±0.19)	0.376	1.000	0.557
**E**	**FPR1: formyl peptide receptor 1**	AA425249	**19q13.4**	**0.44**↓	(±0.25)	0.018	0.261	**0.79**↓	(±0.37)	0.371	1.000	0.436
**E**	**PLCG1: phospholipase C, gamma 1 (formerly subtype 148)**	R76365	**20q12-q13.1**	**0.48**↓	(±0.23)	0.050	0.265	**0.81**↓	(±0.48)	0.656	1.000	0.646
**E**	**ARHGAP8: Rho GTPase activating protein 8**	R69153	**22q13.31**	**0.61**↓	(±0.09)	0.017	0.261	**0.80**↓	(±0.23)	0.307	1.000	0.334
**F**	**REN: renin**	AA455535	**1q32**	**0.44**↓	(±0.32)	0.133	0.269	**0.88**↓	(±0.62)	0.385	1.000	0.326
**F**	**PSMD2: proteasome (prosome, macropain) 26S subunit, non-ATPase, 2**	AA455193	**3q27.1**	**0.72**↓	(±0.39)	0.341	0.448	**0.73**↓	(±0.50)	0.362	1.000	0.280
**F**	**SERPINB6: serine (or cysteine) proteinase inhibitor, clade B (ovalbumin), member 6**	AA410517	**6p25**	**0.72**↓	(±0.13)	0.369	0.477	**0.74**↓	(±0.27)	0.254	1.000	0.193
**F**	**CRYAB: srystallin, alpha B**	AA504943	**11q22.3-q23.1**	**0.50**↓	(±0.08)	0.096	0.265	**0.72**↓	(±0.30)	0.219	1.000	0.199
**F**	**TGM2: transglutaminase 2 (C polypeptide. protein-glutamine-gamma-glutamyltransferase)**	R97066	**20q12**	**0.62**↓	(±0.30)	0.171	0.284	**0.73**↓	(±0.24)	0.182	1.000	0.151
**G**	**C5: somplement component 5**	N73030	**9q32-q34**	**0.74**↓	(±0.09)	0.175	0.286	**0.76**↓	(±0.35)	0.206	1.000	0.122
**H**	**COL5A1: collagen, type V, alpha 1**	R75635	**9q35**	**0.64**↓	(±0.04)	0.618	0.704	**0.78**↓	(±0.28)	0.783	1.000	0.744
**H**	**PNUTL2: peanut-like 2 (Drosophila),**	T64878	**17q22-q23**	**0.81**↓	(±0.15)	0.408	0.514	**1.15**↑	(±0.08)	0.505	1.000	0.520
**H**	**COL18A1: collagen, type XVIII, alpha 1**	W07798	**21q22.3**	**0.57**↓	(±0.17)	0.164	0.279	**0.84**↓	(±0.22)	0.411	1.000	0.414
**I**	**ATP6V0B: ATPase, H+ transporting, lysosomal 21 kD, V0 subunit c"**	AA480826	**1p32.3**	**0.69**↓	(±0.05)	0.213	0.320	**0.84**↓	(±0.26)	0.244	1.000	0.276
**I**	**BZAP45: basic leucine-zipper protein BZAP45**	AA463591	**2q33**	**0.46**↓	(±0.25)	0.151	0.273	**0.73**↓	(±0.30)	0.259	1.000	0.226
**I**	**ATP6IP1: ATPase, H+ transporting, lysosomal interacting protein 1**	AA488715	**Xq28**	**0.44**↓	(±0.32)	0.205	0.312	**0.84**↓	(±0.48)	0.391	1.000	0.315
**J**	**EST**	R00591	**4p16.1**	**3.20**↑	(±1.50)	0.445	0.550	**0.78**↓	(±0.48)	0.455	1.000	0.572
**J**	**RIPX: rap2 interacting protein x**	R74171	**4q21.1**	**0.62**↓	(±0.20)	0.175	0.287	**0.68**↓	(±0.25)	0.251	1.000	0.222
**J**	**EST**	H63361	**12q24.31**	**3.50**↑	(±2.03)	0.271	0.376	**0.66**↓	(±0.26)	0.520	1.000	0.632
**J**	**hypothetical protein BC017488**	W93317	**16q13**	**0.60**↓	(±0.05)	0.006	0.192	**0.80**↓	(±0.24)	0.345	1.000	0.354
**J**	**PTOV1 prostate tumour over expressed gene 1**	AA486332	**19q13.33**	**0.63**↓	(±0.08)	0.090	0.265	**0.84**↓	(±0.17)	0.618	1.000	0.581
**J**	**LOC286467: hypothetical protein LOC286467**	R95805	**Xq26.1**	**2.85**↑	(±1.02)	0.293	0.400	**0.85**↓	(±0.42)	0.530	1.000	0.716

Genes are sorted by functional classification. A: Cellular metabolism (GO:0044237), Metabolism (other than energy metabolism), C: Nucleic acid binding (GO:0003676), D: Transcription regulator activity (GO:0030528), E: Signal transducer activity (GO:0004871), Signal transduction (GO:0007165) and Cell communication (GO:0007154), F: Cellular macromolecule metabolism (GO:0044260), G: Inflammatory response (GO:0006954), H: Organelle organization and biogenesis (GO:0006996I), I: Transport (GO:0006810), J: Biological process unknown (GO:0000004), Upward arrow denotes up-regulated mRNA expression, downward arrow down-regulated mRNA expression, bold indicates genes that have significant alteration in gene expression. Ratio is mean value calculated from three separate microarray experiments; SD is the standard deviation between separate experiments. Statistic probability (p-value) for TGFβ-treated vs. control and for IMR-treated vs. control, were calculated by using t-test. FDR is the calculated Benjamin-Hochberg false discovery rate. All three groups, TGFβ-treated, IMR-treated and control, were compared together with ANOVA.

### Validation of the array data

The genes arrayed twice on the filter acted as internal control points validating the data. The fourteen genes spotted twice on the filter acted as internal control points validating the data. Correlation between the two expression ratios of these genes was good (Pearson correlation coefficient r = 0.673, p = 0.0084), Table 3. Further validation of the data was made with quantitative reverse transcription PCR (RT-PCR) for genes that were selected from different functional classes and from different expression levels from the pool of 372 affected genes (supplementary data list). The expression changes in vaccinia-related kinase 2 (VRK2), ganglioside activator protein (GM2A), crystallin αB (CRYAB), dentatorubral-pallidoluysian atrophy (DRPLA), deiodinase iodothyronine type II (DIO2), proteasome subunit beta type 6 (PSMB), ATP-dependent RNA helicase (ROK1), phospholipase C gamma 1 (PLCG1) and cytokeratin 19 (KRT19) were in concordance with the microarray data, Correlation coefficient between microarray and qRT-PCR results with linear Pearson's correlation was fairly good: r = 0.636, p = 0.0046, Table 4.

**Table 3:** List of genes spotted twice on the microarray filter which evinced a significant change in their mRNA expression in TGFβ-treated T84 cells compared to T84 cells grown solely in collagen I gel (= TGFβ-treated vs. control).

**Gene ontology class**	**Gene name**	**GenBank no**	**chromosomal location**	**TGFβ-treated vs. control**			
				
				**ratio**	**SD**	**ratio**	**SD**
A	FDPS: farnesyl diphosphate synthase (farnesyl pyrophosphate synthetase, dimethylallyltranstransferase, geranyltranstransferase)	T66907 T65907	1q22	0.29↓	(±0.24)	0.57↓	(±0.45)
A	GRP58: glucose regulated protein, 58 kD,	R33030	15q15	0.56↓	(±0.12)	0.60↓	(±0.16)
C	BAP1: BRCA1 associated protein-1 (ubiquitin carboxy-terminal hydrolase)	H09065	3p21.31-p21.2	0.72↓	(±0.19)	0.68↓	(±0.17)
D	DRAP1: DR1-associated protein 1	AA421977 AA406285	11q13.3	0.53↓	(±0.22)	0.60↓	(±0.19)
D	AEBP1: AE binding protein 1	AA490462 AA490684	7p13	0.48↓	(±0.34)	0.60↓	(±0.32)
D	ZNF161: zinc finger protein 161	AA232647	17q23.3	0.71↓	(±0.13)	0.75↓	(±0.11)
E	IGF2: insulin-like growth factor 2 (somatomedin A)	N54596	11p15.5	0.57↓	(±0.16)	0.63↓	(±0.17)
E	MAST1: microtubule associated serine/threonine kinase 1	AA479623	19p13.2	0.51↓	(±0.18)	0.59↓	(±0.48)
E	EGFR: epidermal growth factor receptor (erythroblastic leukemia viral (v-erb-b) oncogene homolog, avian)	R35665 W48713	7p12	0.47↓	(±0.04)	0.75↓	(±0.13)
E	EPHA1: ephrin receptor EphA1	N90246	7q34	0.66↓	(±0.10)	0.63↓	(±0.07)
E	RNTRE: related to the N terminus of tre	AA281057 AA281137	10p13	0.78↓	(±0.25)	0.71↓	(±0.21)
E	EFNB2: ephrin-B2	AA461424 AA461108	13q33	0.77↓	(±0.20)	0.79↓	(±0.06)
F	UBE2N: ubiquitin-conjugating enzyme E2N (UBC13 homolog, yeast)	AA490124	12q21.33	0.76↓	(±0.16)	0.79↓	(±0.17)
I	ATP6V0A1: ATPase, H+ transporting, lysosomal V0 subunit a isoform 1	AA427472	17q21	0.65↓	(±0.02)	0.62↓	(±0.08)

Genes are sorted by functional classification. A: Cellular metabolism (GO:0044237), Metabolism (other than energy metabolism), C: Nucleic acid binding (GO:0003676), D: Transcription regulator activity (GO:0030528), E: Signal transducer activity (GO:0004871), Signal transduction (GO:0007165) and Cell communication (GO:0007154), F: Cellular macromolecule metabolism (GO:0044260), I: Transport (GO:0006810), Downward arrow indicates down-regulated mRNA expression, bold indicates genes that have significant alteration in gene expression. Ratio is mean value calculated from three separate microarray experiments; SD is the standard deviation between separate experiments.

**Table 4:** Microarray data and LightCycler RT-PCR mRNA levels as ratios of VRK2, GM2A, CRYAB, TAGLN, DRPLA, DIO2, PSMB, ROK1, PLCG and KRT19.

**Gene ontology class**	**name**	**GenBank no**	**microarray TGFβ-treated vs. control**		**RT-PCR TGFβ-treated vs. control**		**microarray IMR-treated vs. control**		**RT-PCR IMR-treated vs. control**	
			
			**ratio**	**SD**	**ratio**	**SD**	**ratio**	**SD**	**ratio**	**SD**
A	GM2A	AA453978	0.43↓	(±0.26)	0.39↓	(±0.38)	0.75↓	(±0.47)	0.29↓	(±0.19)
A	DIO2	R62242	0.49↓	(±0.38)	0.01↓	(±0.02)	0.99	(±0.76)	1.12	(±1.6)
D	ROK1	W73792	0.47↓	(±0.25)	0.32↓	(±0.13)	0.80	(±0.47)	1.34↑	(±0.23)
E	DRPLA	H08642	0.65↓	(±0.42)	0.71↓	(±0.12)	0.80↓	(±0.53)	0.66↓	(±0.24)
E	PLCG1	R76365	0.48↓	(±0.23)	0.33↓	(±0.26)	0.81↓	(±0.48)	0.47↓	(±0.28)
F	VRK2	AA490617	0.89↓	(±0.16)	0.04↓	(±0.03)	0.78↓	(±0.22)	0.24↓	(±0.34)
F	CRYAB	AA504943	0.40↓	(±0.08)	0.71↓	(±0.30)	0.72↓	(±0.30)	0.62↓	(±0.13)
F	PSMB	AA070997	0.76↓	(±0.16)	0.30↓	(±0.15)	0.99	(±0.12)	0.51↓	(±0.24)
H	KRT19	AA464250	13.90	(±16.73)	2.43↑	(±0.83)	19.95	(±12.99)	1.14	(±0.32)

TGFβ-differentiated T84 cells compared to T84 cells grown solely in collagen I gel (= TGFβ-treated vs. control) and T84 cells differentiated by soluble factors secreted by mesenchymal cells compared to T84 cells grown solely in collagen I gel (= IMR-treated vs. control). Genes are sorted by functional classification. A: Cellular metabolism (GO:0044237), Metabolism (other than energy metabolism), D: Transcription regulator activity (GO:0030528), E: Signal transducer activity (GO:0004871), Signal transduction (GO:0007165) and Cell communication (GO:0007154), F: Cellular macromolecule metabolism (GO:0044260), H: Organelle organization and biogenesis (GO:0006996I). Upward arrow denotes up-regulated mRNA expression, downward arrow down-regulated mRNA expression, bold indicates genes that have significant alteration in gene expression. Ratios are the mean values calculated from three separate microarray experiments and five separate RT-PCR experiments, the calculated standard deviations (SD) between separate experiments is in the brackets.

### Protein concentrations and immunohistochemistry

**Figure 1:**
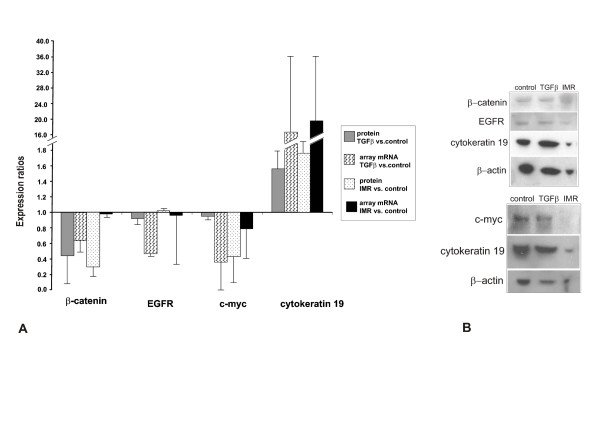
Densitometric analysis of Western blot experiments and microarrays of β-catenin, EGFR, c-myc, cytokeratin 19 protein and mRNA. Proteins and mRNAs were extracted from undifferentiated control cell culture grown in collagen I and medium, or differentiated cultures which were differentiated either by TGFβ1 or soluble factors secreted by IMR-90 cells. Results from the expression ratios from differentiated cell cultures were compared to undifferentiated control culture. The calculated values are from four β-catenin, five cytokeratin 19 two EGFR and two c-myc labeled Western immmunoblots. The EGFR, β-catenin, cytokeratin 1 and c-myc results were from three separate microarrays, EGFR having two separate probes on the filter. Bars indicate the standard deviation of separate experiments (Fig. 1A). Two representative Western blots subjected to densitometric analysis (Fig. 1B).

The Western immunoblotting was carried out with five selected proteins to ascertain whether the changes seen at mRNA level would be in concordance with the protein level. Epidermal growth factor receptor (EGFR) and β-catenin were chosen because they are involved in cell differentiation and we detected changes in their expression at mRNA level. C-myc, a nuclear phospho-protein and oncogene which regulates proliferation in cells was chosen as it has been shown to be up-regulated in proliferating cells [8]. Cytokeratin 19, a protein constructing intermediary filaments, was also chosen because it was highly expressed in differentiated cells [8]. β-actin was used as internal control [18]. The protein expression ratios of β-catenin, EGFR, c-myc and cytokeratin 19 quantitated and calculated from the Western blots (Fig. 1B), were comparable in TGFβ1-differentiated and in IMR-differentiated cells (Fig. 1A).

**Figure 2:**
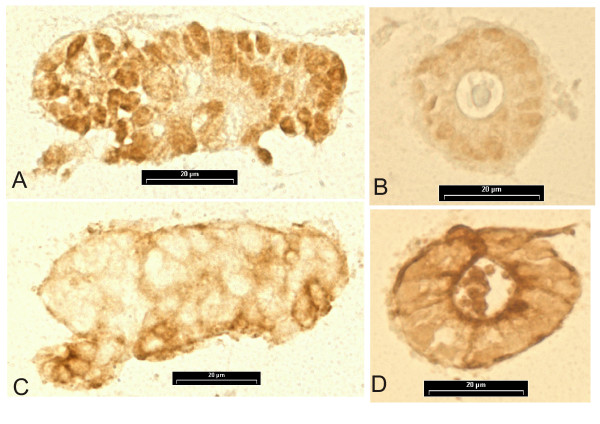
Immunostainings of cell clusters in the three-dimensional intestinal epithelial cell differentiation model. The nuclei in undifferentiated T84 epithelial cell clusters were more intensively labeled with c-myc antibody (Fig. 2A) than nuclei in TGFβ-treated cell clusters (Fig. 2B). The staining intensity with cytokeratin 19 was lower in undifferentiated T84 epithelial cell clusters (Fig. 2C) than in TGFβ-treated cell clusters, especially seen in the apical part of the epithelial cells in the lumen of organized cell cultures (Fig. 2D). Scale bar = 20 μm.

The amount and localization of two selected proteins, c-myc and cytokeratin 19, were further studied with immunostainings. C-myc stained the nuclei intensively in undifferentiated, unorganized T84 epithelial cell clusters (Fig. 2A), whereas in TGFβ-treated, differentiated and organized cell clusters the nuclei labeling was faint (Fig. 2B). In Figures 2C and 2D are the immunostainings with cytokeratin 19. The labeling was clearly up-regulated upon differentiation by TGFβ (Fig. 2D). The immunostainings were in line with the results from microarray and immunoblotting indicating high c-myc and low cytokeratin 19 concentrations in the undifferentiated control culture and low c-myc and high cytokeratin 19 concentrations in TGFβ-differentiated cell culture.

## Discussion

The three-dimensional intestinal epithelial cell differentiation model has been successfully used to study cell differentiation [12-17,19]. In this model crypt-like T84 cells, when grown three-dimensionally in collagen gel, are induced to differentiate either by the soluble factors secreted from IMR fibroblasts or by TGFβ1 [12,13]. IMR-induced differentiation has been shown to occur in a TGFβ-dependent manner [12], and it has been concluded that the differentiation of T84 cells in the present co-culture models the differentiation of epithelial cells in intestine [12,13,19].

Here we used the cDNA microarray method and our cell culture model to gain additional insight into the genetic programming of epithelial cells upon differentiation. Furthermore, we examined similarities and differences in gene expressions when differentiation was induced either by TGFβ1 or by mesenchymal cell (IMR-90 fibroblast) soluble factors. Differentiation altered expression of 372 genes, and 47 of them were altered by both mediators.

A major trend seen in differentiated cultures was a diminishing expression of transcription factors, growth factors and molecules regulated by them. Also the transcription of genes attending to the mRNA processing, translation and folding of proteins were decreased in TGFβ1-differentiated epithelial cells. The decreased expression of the above-mentioned factors and signaling molecules, such as cathepsin S or MRLP, were similar in our three-dimensional epithelial cell differentiation model to those observed in spontaneously differentiating Caco-2 adenocarcinoma cells [8-10,18,20]. Also in the mouse cell lines, the genes attending to proliferation and transcription regulation, for example c-myc, have been shown to decrease during differentiation of epithelial cells [9,21,22]. In other studies of epithelial differentiation, both in vivo [9] and in vitro [10,18], the number of down-regulated genes compared to up-regulated genes has not been so prominent as in this study. However, it is noteworthy that in these previous studies, as well as in our study, most functional groups show particularly distinct down-regulation of gene expression in differentiated cells.

There were several genes that are previously shown to be affected in spontaneously differentiating Caco-2 cells, and that had significant change transcription in our model: The transcription of genes associated with proliferation such as c-myc or cyclin D2 were decreased in TGFβ1-differentiated cells and in differentiated Caco-2 cells [8,10,18]. Also the genes coding proteins attending to the gene expression, for example helicases and zinc finger proteins, were down-regulated in TGFβ1- and IMR-differentiated cells similarly as in differentiated Caco-2 cells [18]. Transcription of genes associated to protein synthesis, such as ribosomal proteins and translation initiators (E2F4) [10], were decreased in TGFβ1- and IMR-differentiated as in differentiated Caco-2 cells [10,18]. Decreased expression of ubiquiting-conjugating enzymes was consistent with the findings derived from differentiated Caco-2 cells [10,18]. Ubiquitinated proteins then recognized by 26S proteasome complex and then destructed. From proteasome complex proteins one component of 26S complex (PSMD2) and two components of 20S complex (PSMA3 and PSMB6) were down-regulated in TGFβ1-differentiated cells. In differentiated Caco-2 cells Mariadason et al. did not find alterations in 26S complex but they found several components of 20S complex being down-regulated [18]. In that study expression of several signaling molecules in MAPK-pathway were decreased [18], whereas in our study only the expression of MAPKAPK2 from MAPK-pathway was significantly decreased. Genes that were commonly affected in TGFβ-, wnt- and receptor tyrosine kinase-signaling routes are being discussed later in the text.

In cell division and cellular growth there is an increase in expression of genes regulating transcription, as well as genes coding for mRNA splicing machinery, protein translation and protein folding machinery [22]. We saw an up-regulation of genes that control protein synthesis such as ribosomal protein (RPS5), mitochondrial ribosomal protein (MRLP4), and initiation factor (EIF2B2) in undifferentiated cells. Genes taking part to nuclear RNA processing (PRP18) and splicing, (SFRS1 and SF3A1), exhibited up-regulation in undifferentiated epithelial cells compared to the differentiated cells. Also genes coding ubiquiting-conjugating enzymes (UBE2N and UBE2I), and two genes from proteasome complex (PSMA3 and PSMB6), were up-regulated in undifferentiated epithelial cells. Increased demand of energy in proliferating cells in known to induce up-regulation of genes coding oxidative phosphorylation machinery [22]. In our model genes coding components of oxidative phosphorylation pathway, (SDHB and UQCR), were up regulated in undifferentiated cultures. All these above mentioned genes, were up-regulated in dividing epithelial cells in the study of Stappenbeck et al. that was done with mouse small intestinal epithelial progenitors [22]. Hence these changes in the gene expression imply that epithelial cells grown untreated in collagen I gel cultures were more actively dividing than epithelial cells treated with TGFβ1 or with IMR fibroblasts.

### Genes involved in TGFβ-, wnt- and receptor tyrosine kinase-signaling routes (Fig. 3)

**Figure 3:**
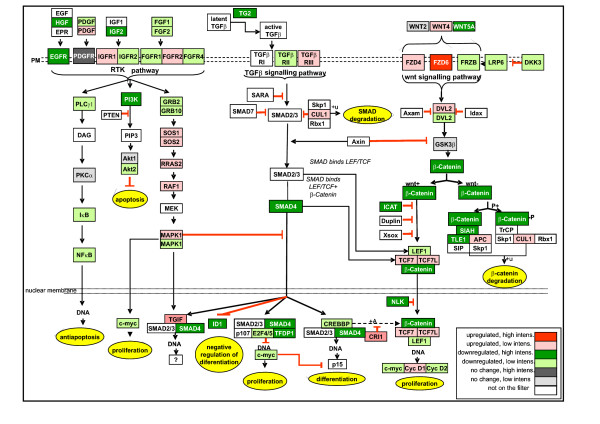
Schematic illustration of mRNA expression levels in TGFβ-treated T84 epithelial cells compared to control cell culture. Results are from three separate experiments. In TGFβ-treated cells the expressions of EGFR, PI3K, c-myc and GRB in the Receptor tyrosine kinase (RTK) pathway were decreased. In the TGFβ signaling pathway the expression of TFDP1 and E2F4/5 were decreased, TGIF increased and c-myc as well as IDs decreased. In the TGFβ-treated cells transcription of β-catenin was decreased in the wnt signaling pathway, the wnt+-route was down-regulated, as indicated by down-regulation of c-myc and cyclin D2. The red indicates up-regulation in mRNA expression in a gene with high expression level; pink indicates up-regulation in gene with a low expression level. Dark green indicates a significant down-regulation in a gene which has high expression level; light green indicates down-regulation in a gene with low expression level; dark grey indicates a high level of expression with no change, light grey no change with low concentration expression. White indicates absence from the filter.

The transcription of the genes on TGFβ-, receptor tyrosine kinase-(RTK), and wnt-pathways are known to be affected upon differentiation of the epithelial cells [8-11,18,23]. As our epithelial cell differentiation model revealed a number of both expected and new transcripts regulated by TGFβ which might be involved in these pathways, all results from microarrays were depicted in the form of signaling cascades. The detailed signaling pathway picture (Fig. 3) was drawn according to the Kyoto Encyclopedia of Genes and Genomes (KEGG) [24] in homo sapiens [25] and according to previously published results [2-4,26-37]. The focus was set to describe the cross-talk between signaling pathways.

In our epithelial cell differentiation model TGFβ1 induced up-regulation of TGFβ co-repressor TGIF and TGFβ early growth response gene (TIEG), and down-regulation of type II TGFβ receptor (Tβ RII), CREB binding protein (CREBBP), transcription factor Dp-1 (TFDP1), E2F4/5, c-myc and Id1 (Fig. 3). In the intestine TGFβ receptors and ligands are expressed predominantly in the differentiated compartment [1]. In Caco-2 cells the expression of Tβ RII has been shown to spike during transition from proliferation to differentiation and then to decline after transition [10]. In Caco-2 cells the induction of TIEG transcription is used as an indicator of activation of the TGFβ-signaling pathway by differentiation [10]. In another study made with Caco-2 cells the transcription of E2F5 and c-myc was shown to decrease upon differentiation [18]. Comparing our results to others, it appeared that the TGFβ pathway was active in our TGFβ-treated, differentiated, cell culture [8,10,18,27].

We showed that TGFβ1 induced down-regulation of the expression of β-catenin, SIAH, ICAT and TLE1. However, E-cadherin remained constant and the expression of Tcf-4 was exiguous in the wnt pathway (Fig. 3). The wnt-signaling pathway is known to abate in differentiated epithelial cells in cultures [8,10,18] and in the intestine [9,20]. It would thus appear that the wnt signaling pathway down stream on β-catenin is quiescent in TGFβ-differentiated cells [8-10,18,20].

Furthermore TGFβ1 decreased expression of the EGFR, the p85 subunit of PI3-K, PLCγ, c-myc, GRB and NFκB (Fig. 3). In normal epithelial cells the activation of the RTK-pathway, for example EGFR, is known to activate PI3-kinase and PLCγ [33] and also to promote proliferation and growth [33,38]. The decreased activation upstream of the RTK-pathway might be a result of decreased expression of c-myc and decreased proliferation [33]. The expression of the p85 subunit of PI3-K was decreased in our TGFβ-differentiated T84 cells. In contrast to our results, in differentiated Caco-2 cells the transcription of PI3-K p85 has been found to be increased [10]. However, the over-expression of PI3-K p85 subunit is shown to inhibit differentiation and over-expression of p110 to promote differentiation [39], suggesting that a balance between different subunits is vitally important.

In the present study TGFβ1-induced differentiation appeared to alter the expression of the signaling molecules upstream on the RTK- and wnt-pathways. We therefore directed our attention to the mechanisms underlying the reprogramming events taking place during epithelial cell differentiation. In order to understand the mechanisms underlying the differentiation of epithelial cells it is essential to understand precisely how the signals are modulated and fine-tuned within the cells. During the differentiation of epithelial cells several signaling pathways are known to act synergistically and to interact via common mediators, modulate the outcomes by varying the interacting partners [28,29,31,33,35,36], or co-operatively fine-tune the transcription of a single molecule [2,23,27,32-34,40]. For example Akt, a serine/threonine kinase, is a molecular nodal point of several signaling cascades [36] which can deliver the signal to several different pathways and alter the outcome of the signal as well: In the wnt-pathway Akt activates proliferation by preventing the formation of the GSK3β/APC/β-catenin-destruction complex [33], in the NFκB route it regulates antiapoptosis [35], and by positively regulating CREBBP it also regulates differentiation [36]. In our three-dimensional epithelial cell differentiation model in TGFβ1-treated cells Akt2 was down-regulated (Fig. 3). Thus the decreased expression of Akt may induce the down-regulation of the wnt and NFκB-route and decreased signaling to CREBBP. Indeed all these signaling routes (Fig. 3.) were down-regulated in TGFβ1-treated culture. Several signaling pathways can synergistically modulate the function of a single gene such as c-myc, whose expression is tightly regulated by the TGFβ-, wnt- and RTK-pathways. Activation of the TGFβ route reduces expression of c-myc [27] contribute to growth arrest [32], whereas activation of the wnt and RTK-pathways induces c-myc expression and proliferation [23,33,40]. C-myc has been shown to be down-regulated upon differentiation in epithelial cell cultures [8,10,18] and in the normal intestine [9,40]. In our crypt villus axis model we saw down-regulation of c-myc at both mRNA and protein levels in differentiated cells, suggesting the synergistic action of these signaling routes to the expression of c-myc (Fig. 3). It appears that the molecular changes seen in RTK- and wnt-signaling pathways are consequences of TGFβ1. Our results suggest that TGFβ1 modulates, either directly or indirectly, the transcription of the mediators on these signaling pathways (Fig. 3).

T84 cells have been reported to respond correctly to added stimulants in the TGFβ- and RTK-pathways, and the wnt-pathway at β-catenin level [12,13,41-43]. As all cancerous cell lines are known to have mutations in their genes [44], the molecules under investigation might also have been mutated [43] and this must be borne in mind when interpreting results.

### Differentially expressed novel, as yet uncharacterized genes

Altogether 26.5% of the differentially expressed genes detected here had hitherto no characterized functions. Several of them for example LOC440582, RP4-622L5, FLJ12057, FCHO2, SMG1 and several ESTs (Table 1.) are reported to be expressed at the embryonic stage but not in juveniles. Furthermore they have been shown to be expressed in the intestine [51]. These sequences with unknown function are especially interesting in the search for new players which may act in epithelial cell differentiation.

### TGFβ-induced versus IMR fibroblast-induced differentiation

The comparison of results from TGFβ1- and IMR-differentiated cultures revealed 47 commonly altered genes. Majority of them [39] were similarly expressed, down-regulated. It is known that mesenchymal cells secrete several soluble factors which control epithelial growth, motility and morphogenesis [6]. IMR fibroblasts, by secreting several mediating factors, induce natural differentiation [1,45]. The IMR fibroblast-induced differentiation of T84 cells has been shown to occur in a TGFβ1-dependent manner [12]. However, after seven days of culturing a smaller proportion of cells is differentiated in IMR-differentiated than in TGFβ1-differentiated culture [12]. Also in the present study, the expression changes detected by microarray were stronger in the TGFβ1-differentiated cells, compared to IMR-differentiated cells. In Caco-2 cells the expression of platelet-derived growth factor receptor (PDGFR) has been shown to spike during transition from proliferation to differentiation and to decline after the transition [10]. In our model the transcription of PDGFR was up-regulated in IMR- but unchanged in TGFβ-differentiated cells (Table 4). Comparing our results to the ones from Caco-2 cells, it looks as IMR-differentiated cells may have been in transition from proliferation to differentiation and TGFβ1-differentiated cell cultures had already passed the transition stage. According to the mRNA expression data, the TGFβ1-induced differentiation appears to be more potent than the natural IMR fibroblast-induced differentiation. This might reflect the fact that TGFβ1 is a single, potent differentiating factor always inducing similar differentiation, whereas the IMR fibroblasts secrete various factors which may induce variation also in differentiation.

## Conclusion

Our results suggest that TGFβ1 modulates mediators on wnt- and receptor tyrosine kinase-pathways. By comparing mRNA expression patterns from TGFβ1-differentiated epithelial cells and mesenchymal cell soluble factor differentiated epithelial cells the differentiation induced by TGFβ1 might be more potent than the differentiation induced by mesenchymal cells. This study indicates that this three-dimensional epithelial cell differentiation is a suitable tool in studying regulatory mechanisms during differentiation in intestine. In conclusion the present results would indicate that our model is a good tool for finding new players acting in the differentiation of epithelial cells.

## Methods

### Sample material

#### Cell lines and cell cultures

Human intestinal epithelial T84 cells (CCL 2'48, ATCC Rockville, MD, USA) were cultured in three-dimensional type I collagen gel as previously described [12]. T84 cells were induced to differentiate either by adding 20 ng/ml human recombinant TGFβ1 (hTGF-β1, R&D Systems Europe, Oxon, UK) or by soluble factors secreted by IMR-90 type human embryonic lung fibroblasts (CCL 186, ATCC). IMR fibroblasts, cultured on the top of the epithelial cells, were separated from the epithelial cells by cell-free collagen layer. T84 cells cultured within collagen gel supplemented with medium were used as undifferentiated control. We studied only undifferentiated and differentiated epithelial cells harvested after seven days of culturing. All experiments were carried out in triplicate.

### Isolation of RNA

The mRNA was extracted from the cell culture samples to ice-cold TRIzol reagent (Life Technologies, Inc. Frederick, MD, USA) according to the manufacturer's protocol. All samples were subjected to DNAse I treatment (Roche Diagnostics GmbH, Mannheim, Germany). Purity and quantity of total RNA was determined by spectrophotometry (Bio Rad, Sweden) and quality was checked by agarose gel electrophoresis. The amount of rat tail collagen was same both in undifferentiated and differentiated cultures, therefore potential rat RNA contamination would not produce a problem in microarray analysis.

### cDNA synthesis and array hybridization

Gene expression was monitored using a Human GeneFilter GF200 (Research Genetics, Huntsville, AL, USA), consisting of 5188 test sequences, 96 control points and 192 housekeeping genes. Arrayed sequences contained both genes with known or predicted function and expressed sequence tags (ESTs) with unknown function. Probe preparation and microarray hybridization were performed following manufacturer's (Research Genetics, Huntsville, AL, USA) protocol using 1.5 μg total RNA as template, 10 μl (10 mCi/ml) ^33^P dCTP (ICN Radiochemicals), 1,5 μl dNTP mix containing dATP, dTTP, dGTP at 20 mM (Finnzymes, Finland), 1,5 μl reverse transcriptase (Life Technologies, Inc. Frederick, MD, USA), 1,0 μl DTT (Life Technologies, Inc. Frederick, MD, USA). ^33^P-labeled cDNAs were synthesized for 90 min at 37°C. ^33^P-cDNA products were separated from unincorporated nucleotides by chromatography on Bio-Spin 6 (Bio-Rad, Sweden). Filter was prehybridized with human 5 μg Cot-1 (Life Technologies, Inc. Frederick, MD, USA) DNA and 5 μg poly dA (Research Genetics, Huntsville, AL, USA) in MicroHyb solution (Research Genetics, Huntsville, AL, USA) to minimize non-specific labeling. The denatured ^33^P-cDNA was added and incubated for 12 h at 42°C in roller bottle. The arrays were washed at high stringency (twice in 2 × SSC 1%SDS, and 0.5 × SSC) and signals were detected from storage phosphor screens by Storm 860 phosphoimager (Molecular Dynamics, Amersham Biosciences, Buckinghamshire, England) with 50-micron resolution as previously described [46]. The label was removed from filter by boiling in 0.5% SDS. The efficiency of the removal was ensured with storage phosphor screens by Storm 860 phosphoimager after exposing for 12 h. The undifferentiated, TGFβ1-differentiated and IMR-differentiated samples were hybridized on same filter. All the experiments were done in triplicate.

### Processing and statistical analysis of the microarray data

Filter images were aligned and spot intensities analyzed with Pathways Software (Research Genetics). The handling of the raw data, normalization and improvement of fidelity by setting a cut-off value were done as previously described in Juuti-Uusitalo and associates [46]. Intensity values were normalized in order to avoid possible differences in the amounts of RNA and differences in hybridization efficiency. The normalization factor was determined before background subtraction. The background intensity was determined by the Pathways Software (Research Genetics). The normalization was done by the sum method [47]. The scaling factor was set to ensure that the sums of spot intensities were equal for all filters. The scatter plots for the normalized data are shown as the Additional data (see Additional file 1 and Additional file 2).

Prior to statistical analyses the data normalized by the sum method was further normalized using a linear mixed model. Normalizing data using a mixed model effectively removes possible filter effects from the data. Mixed models contain both fixed effects that were manipulated during the study and random effects that were not or could not be manipulated during the study. In the current setting, the best performing models treated cell lines and filters as fixed effects, and the hybridization order on every filter as a random effect. Models with and without interaction between fixed and random effects were compared using analysis of variance, and were found not to differ significantly. Therefore, models without interaction terms were fitted. A separate model was fit for every gene. Residuals from the models were approximately normally distributed and were used for statistical analyses. Two population t-test was used for comparing gene expression of differentiated cells to undifferentiated. The t-test p-values and Benjamin-Hochberg false discovery rates (FDR) are shown in Tables 1 and 2. The linear mixed modeling and statistical analyses were performed using R software, version 2.3.1., and its library nlme version 3.1–73.

Approximately 11% of the genes were below the average background intensity. Mills and Gordon by using oligonucleotide arrays, have demonstrated that false positive values were more frequent at the lowest level of expression [48]. Therefore, in order to improve fidelity, the low intensity genes, 85% of all genes, were removed by setting a cut-off value. Only genes with a low spot intensity value in both of the compared pairs were removed.

According to Lee and Whitmore [49] three replicates are necessary to obtain consistent and reliable findings in cDNA microarray. Therefore we had three replicates, and genes that had the change to same direction in all three replicates we would get the result that is true for the whole experimental group.

In order to eliminate the variation arising from the culture conditions such as passage number and collagen support, only those samples cultured, treated and extracted at the same time and hybridized on the same filter were compared with each other. Furthermore, only those genes evincing the change in the same direction in all sample pairs within a sample group and the mean ratio which was above 1.25 were considered significant. 372 genes or ESTs fulfilled these criteria and their mRNA expression was considered to be significantly altered. As we took for analysis only those genes that were altered in all sample pairs within a sample group, we aimed to focus to the genes actually altered in the whole sample group.

Clustering of genes was done with software programs Cluster and Treeview and R 2.3.1 (package gregmisc version 2.0.8). Prior to hierarchical clustering data was log2 transformed and expression values on all chips were translated to the same mean (0). Also, the residuals from mixed models were clustered to check that the filter specific effect was removed. Spearman rank correlation coefficient was used for calculation of distances between genes or experiments. The hierarchical clustering tree was inferred from the distances using average linkage method. Cluster analysis for unfiltered data is presented as additional file (see Additional file 3) and the cluster analysis for the residuals from mixed models is presented as additional file (see Additional file 4). This data shown that, as anticipated, the filter specific effect can be removed and the comparisons of the expression values effectively performed.

The genes were grouped manually into ontology classes according their known or predicted functions defined in Gene Ontology Consortium [50]: Genes were sorted by functional classification. A: Cellular metabolism (GO:0044237), Metabolism (other than energy metabolism), B: Generation of precursor metabolites and energy GO:0006091), C: Nucleic acid binding (GO:0003676), D: Transcription regulator activity (GO:0030528), E: Signal transducer activity (GO:0004871), Signal transduction (GO:0007165) and Cell communication (GO:0007154), F: Cellular macromolecule metabolism (GO:0044260), G: Inflammatory response (GO:0006954), H: Organelle organization and biogenesis (GO:0006996I), I: Transport (GO:0006810), J: Biological process unknown (GO:0000004). The sequences on the arrays were not annotated by Research Genetics, as the filter was released in 1996. Therefore the annotation for the significantly altered genes was done as follows: Sequence was sought with the UniGene code given by Research Genetics from Entrez UniGene [51] site that had the direct link to NCBI Entrez Gene [52] that contains information about genomic annotation and information about ontologies. In this study annotations were done according to the NCBI Entrez Gene [52]. The ontologies on that site were supplied by EMBL-EBI GOA [53].

Genes which were arrayed twice on the GF200 filter functioned as internal control points. The microarray experiments and data included in this manuscript are available in Gene Expression Omnibus repository at National Center for Biotechnology Information [54] as Accession GSE5170.

### Real-time RT-PCR

Confirmation of microarray results was made with real-time quantitative reverse transcription PCR (RT-PCR) as previously described [46]. Primers were designed with the assistance of the Primer 3 program [55] and were chosen according to the requirements previously presented [46]. Genes for confirmation were selected from different functional classes. The intensity values of the validated genes varied from high (PSMB), to average (PLC6, EGFR and β-catenin), below average (GM2A, CRYAB and DRPLA) to intensity values just above the cut off threshold (DIO2, ROK1) and below the cut off threshold (KRT19). The probe sequences for primers and their functional classes are set out in Table 5. PCR reactions were carried out in the LightCycler apparatus using the LightCycler-FastStart DNA Master SYBR Green I Kit (Roche Diagnostics GmbH) as previously described [46]. The expression levels of VRK2, GM2A, CRYAB, DRPLA, DIO2, PSMB, ROK1, PLCG1 and KRT19 were measured by quantitative PCR and were normalized by the expression values of housekeeping gene GAPDH. After PCR, every sample was also run in 1.5% agarose gel electrophoresis to ensure that a product of correct size was amplified in the reaction.

**Table 5:** List of used primers, their temperature and MgCl optimum and length of product.

name	direction	sequence	size	MgCl μmol	annealing temp
GAPDH	F	ATG CCA GTG AGC TTC CCG TTC AGC	199	2	70
GAPDH	R	TGG TAT CGT GGA AGG ACT CAT GAC			
VRK2	F	GCAGAAAGAGGAGAAACTGATTGGA	245	4	62
VRK2	R	CCGTGCTGACTGTGGAAGTGTATT			
GM2A	F	TTCCTTGCCACTGTCCCTTCA	222	3	61
GM2A	R	CTTCCTCACACCGCTCCATTCT			
CRYAB	F	CCCCTTCTTTCCTTTCCACTCC	266	3	62
CRYAB	R	CACCTCAATCACATCTCCCAACAC			
DRPLA	F	GAACTCTCCCTAACCCCCTGCTT	279	3	63
DRPLA	R	GTGGCTTGTCGCTTTCCTTCTTC			
DIO2	F	GGGCATCCTCAGCGTAGACTTG	295	4	64
DIO2	R	GCCACTGTTGTCACCTCCTTCTGT			
PSMB	F	TATTTATTGTTGTGGTGCTGGGACA	350	4	65
PSMB	R	TCTTGGCTTCCTCCTCCTCCA			
ROK1	F	CCTGTTCTTGTTTTTGTTCAGTCCA	298	3	61
ROK1	R	TTGCTTTTCCCTTATTCCCTGCTC			
PLCG1	F	CTCAACTTCCAGACCCCTGACAA	237	3	64
PLCG1	R	CACCTCAATCTCCACAAAAGGACAC			
KRT19	F	GCACCCTTCAGGGTCTTGAGAT	346	3	64
KRT19	R	AAGACACCCTCCAAAGGACAGC			

### Protein extraction and Western blotting

For Western immunoblotting proteins from the three-dimensional cell culture were extracted directly to Laemmli buffer [56]. Epithelial cells in the three-dimensional cell culture were grown within collagen I gel, thus containing an excessive amount collagen protein. Loading to the gel was therefore done by approximating the number of cells in cultures. All samples were denatured and run in Tris-Glycine Precast Gels (Invitrogen, Carlsbad, CA, USA). Proteins were blotted according to the manufacturer's protocol (Invitrogen) to the nitrocellulose filter (Hybond C-Extra, Amersham Biosciences, Ltd.). Polyclonal antibodies against epidermal growth factor receptor (EGFR), cytokeratin 19 and c-myc (Santa Cruz Biotechnology, Inc. CA, USA.) and β-catenin (BD Transduction Laboratories, CA, USA) and a monoclonal antibody against β-actin (Sigma-Aldrich, Saint Louis, MO, USA) were used for immunoblotting. The secondary antibody anti goat-HRP-conjugate (DAKO, Glostrup, Denmark) was for detecting EGFR, anti rabbit-HRP-conjugate (DAKO) for c-myc, anti-mouse-HRP-conjugate (DAKO) for β-catenin, cytokeratin 19 and β-actin. The nitrocellulose filter was incubated in EGFR or c-myc primary antibody dilution and a comparable secondary antibody dilution, and then in ECL Plus Western Blotting Detection Reagent (Amersham Biosciences Ltd.), and finally exposed on Hyperfilm (Amersham Biosciences, Ltd.) according to the ECL Plus Western Blotting Detection Reagent (Amersham Biosciences, Ltd.) protocol. Primary and secondary antibodies were removed from the filter according to manufacturer's protocol. The filters were then re-probed with other primary antibodies and processed as described above. The relative amounts of β-catenin, cytokeratin 19, EGFR and c-myc were calculated from scanned images (grey-scale, 1200 dpi, TIF) with Amersham Image Quant TL (Amersham Biosciences, Ltd.). The calculated values are from four β-catenin, five cytokeratin 19, two EGFR and two c-myc labeled Western blots.

### Immunohistochemical studies

In order to verify immunoblotting results TGFβ-differentiated cultures, IMR-differentiated and control cultures immunostained with cytokeratin 19 and c-myc antibodies (Santa Cruz Biotechnology, Inc.) as previously described [14]. The protocol in detail: After one week of culturing, the cells were washed twice in PBS. They were fixed with 10% formalin over night at room temperature. Cell cultures were processed in Shandon Citadel 1000 (Thermo electron corporation, MA, USA) tissue processor where they were dehydrated once for 15 min in 70% ethanol, once for 15 min in 96% ethanol, three times for 15 min in 100% ethanol, three times for 15 min in xylene) and paraffined twice for 30 min in melted paraffin. Finally the samples were embedded to the paraffin blocks. 5 μm sections were cut from paraffin blocks. After the deparaffination of tissue sections three times for 10 min in xylene, hydration (three times for 5 min in 99% ethanol, three times for 3 min in 94% ethanol, once for 5 min in PBS) then the antigen retrieval was performed by boiling slides for 10 min in 0.01 M citrate buffer (pH 6.0), followed by cooling to room temperature. Three washes in PBS (5 min each) and blocking of the non-specific binding sites in normal horse serum for 1 h at room temperature. Sections were subsequently incubated overnight at +4°C with either cytokeratin 19 or c-myc the primary antibody. After overnight incubation at +4°C sections were washed three times for 5 min in PBS, incubated in secondary antibody for 30 min at room temperature, and the washed twice for 5 min in PBS. Endogenic peroxidase activity was removed with 0.3% H_2_O_2_, for 1 h at room temperature. ABC-reaction was done with the Vectabond TM reagent (Vector Laboratories, Inc. Burlingame, CA, USA) 30 min at room temperature. Sections were washed twice for 5 min in PBS. The peroxidase reaction was performed for 5 min at room temperature using DAB (DacoCytomation Inc. CA, USA) as precipitate forming substrate. Finally the sections were washed washed with tap water, dehydrated and mounted to Mountquick (Daido Sangyo Co. Ltd, Tokyo, Japan). Sections were left without counterstain to highlight the labeling pattern. Sections not incubated with primary antibodies served as negative controls.

## Authors' contributions

K.J-U conducted all experimental procedures including array experiments, data mining, and selected genes of interest, verified results with quantitative RT-PCR, did immunoblotting, immunohistochemical staining and drafted the manuscript. K.K participated in study coordination, drafted the manuscript and edited visual appearance. M.M. created the original study design, drafted the manuscript and edited visual appearance. J.T carried out the statistical analysis and drafted the manuscript. H.K. participated in study design and drafted the manuscript. All authors read and approved the final manuscript.

## Supplementary Material

Additional file 5 List of genes evincing a significant change in their mRNA expression in TGFβ-differentiated compared to T84 cells grown solely in collagen I gel (= TGFβ vs. control) and T84 cells differentiated by soluble factors secreted by mesenchymal cells compared to T84 cells grown solely in collagen I gel (= IMR-treated vs. control). Genes are sorted by functional classification. A: Cellular metabolism (GO:0044237), Metabolism (other than energy metabolism), B: Generation of precursor metabolites and energy (GO:0006091), C: Nucleic acid binding (GO:0003676), D: Transcription regulator activity (GO:0030528), E: Signal transducer activity (GO:0004871), Signal transduction (GO:0007165) and Cell communication (GO:0007154), F: Cellular macromolecule metabolism (GO:0044260), G: Inflammatory response (GO:0006954), H: Organelle organization and biogenesis (GO:0006996I), Transport (GO:0006810), J) Biological process unknown (GO:0000004), Upward arrow denotes up-regulated mRNA expression, downward arrow down-regulated mRNA expression, bold indicates genes that have significant alteration in gene expression. Ratio is mean value calculated from three separate microarray experiments; SD is the standard deviation between separate experiments. Statistic probability, p-value, was calculated by using t-test. FDR is the calculated Benjamin-Hochberg false discovery rate. Click here for file

Additional file 1 The scatter plot for the normalized data: TGFβ-differentiated compared to undifferentiated T84 cells grown solely in collagen I gel. Click here for file

Additional file 2 The scatter plot for the normalized data:T84 cells differentiated by soluble factors secreted by mesenchymal cells compared to undifferentiated T84 cells grown solely in collagen I gel. Click here for file

Additional file 3 Cluster analysis for unfiltered data. Clustering of genes was done with software programs Cluster and Treeview. Click here for file

Additional file 4 Cluster analysis, heatmap, for the data first normalized by the sum method and further normalized using a linear mixed model. The Clustering of genes was done with software programs Cluster and Treeview. Click here for file
