# Supplementary material for: Gene expression in TGFbeta-induced epithelial cell differentiation in a three-dimensional intestinal epithelial cell differentiation model
Source: BMC Genomics. 2006 Oct 31;7:279. doi: 10.1186/1471-2164-7-279 (PMC1635984; doi:10.1186/1471-2164-7-279)
Supplement: Additional file 5 — List of genes evincing a significant change in their mRNA expression in TGFβ-differentiated compared to T84 cells grown solely in collagen I gel (= TGFβ vs. control) and T84 cells differentiated by soluble factors secreted by mesenchymal cells compared to T84 cells grown solely in collagen I gel (= IMR-treated vs. control). Genes are sorted by functional classification. A: Cellular metabolism (GO:0044237), Metabolism (other than energy metabolism), B: Generation of precursor metabolites and energy (GO:0006091), C: Nucleic acid binding (GO:0003676), D: Transcription regulator activity (GO:0030528), E: Signal transducer activity (GO:0004871), Signal transduction (GO:0007165) and Cell communication (GO:0007154), F: Cellular macromolecule metabolism (GO:0044260), G: Inflammatory response (GO:0006954), H: Organelle organization and biogenesis (GO:0006996I), Transport (GO:0006810), J) Biological process unknown (GO:0000004), Upward arrow denotes up-regulated mRNA expression, downward arrow down-regulated mRNA expression, bold indicates genes that have significant alteration in gene expression. Ratio is mean value calculated from three separate microarray experiments; SD is the standard deviation between separate experiments. Statistic probability, p-value, was calculated by using t-test. FDR is the calculated Benjamin-Hochberg false discovery rate. [file 1471-2164-7-279-S5.doc]

**Additional file 5.:** List of genes evincing a significant change in their mRNA expression in TGFβ-differentiated compared to T84 cells grown solely in collagen I gel (= TGFβ vs. control) and T84 cells differentiated by soluble factors secreted by mesenchymal cells compared to T84 cells grown solely in collagen I gel (= IMR-treated vs. control).

| A: Cellular metabolism GO:0044237  Metabolism (other than energy metabolism) |  |  |  | | | |  | | | |
| --- | --- | --- | --- | --- | --- | --- | --- | --- | --- | --- |
| Gene name | GenBank no | chromosomal location | TGFβ−treated vs. control | | | | IMR-treated vs. control | | | |
|  |  |  | ratio | SD | p-value | FDR | ratio | SD | p-value | FDR |
| GSTM4: glutathione S-transferase M4 | AA486669 | 1p13.3 | **0.57**↓ | (±0.11) | 0,123 | 0,265 | 1.12 | (±0.66) | 0.980 | 1.000 |
| GSTM2: glutathione S-transferase M2 (muscle) | AA290737 | 1p13.3 | **0.74**↓ | (±0.26) | 0,121 | 0,265 | 1.04 | (±0.02) | 0.751 | 1.000 |
| PGM1: phosphoglucomutase 1 | AA488504 | 1p31 | **0.58**↓ | (±0.28) | 0,234 | 0,345 | 1.16 | (±0.67) | 0.945 | 1.000 |
| DHCR24: 24-dehydrocholesterol reductase | AA482324 | 1p33-p31.1 | **0.68**↓ | (±0.20) | 0.699 | 0.733 | 1.01 | (±0.14) | 0.969 | 1.000 |
| FDPS: farnesyl diphosphate synthase (farnesyl pyrophosphate synthetase, dimethylallyltranstransferase, geranyltranstransferase) | T66907  T65907 | 1q22 | **0.29**↓  **0.57**↓ | (±0.24)  (±0.45) | 0.180  0.148 | 0.291  0.272 | 1.43  1.06 | (±1.32)  (±1.02) | 0.807  0.499 | 1.000  1.000 |
| CYP1B1: cytochrome P450, family 1, subfamily B, polypeptide 1 | AA448157 | 2p21 | **0.66**↓ | (±0.26) | 0.724 | 0.795 | **0.68**↓ | (±0.23) | 0.716 | 1.000 |
| PIG3: quinone oxidoreductase homolog | AA668595 | 2p23.3 | **0.64**↓ | (±0.09) | 0.218 | 0.325 | 1.31 | (±0.52) | 0.288 | 1.000 |
| UMPS: uridine monophosphate synthetase (orotate phosphoribosyl transferase and orotidine-5'-decarboxylase) | AA426227 | 3q13 | **0.65**↓ | (±0.56) | 0.365 | 0.464 | **0.68**↓ | (±0.45) | 0.270 | 1.000 |
| APOD: apolipoprotein D | H15842 | 3q26.2-qter | **0.51**↓ | (±0.14) | 0.036 | 0.265 | 1.06 | (±0.57) | 0.828 | 1.000 |
| DCK: deoxycytidine kinase | H12903 | 4q13.3-q21.1 | 0.94 | (±0.28) | 0.891 | 0.926 | **1.32**↑ | (±0.18) | 0.244 | 1.000 |
| ADH6: alcohol dehydrogenase 6 (class V) | H68509 | 4q23 | **0.58**↓ | (±0.32) | 0.499 | 0.598 | 0.99 | (±0.39) | 0.675 | 1.000 |
| ALDH7A1: aldehyde dehydrogenase 7 family, member A1 | AA101299 | 5q31 | **0.57**↓ | (±0.10) | 0.178 | 0.289 | 0.95 | (±0.17) | 0.677 | 1.000 |
| GM2A: GM2 ganglioside activator protein | AA453978 | 5q31.3-q33.1 | **0.36**↓ | (±0.26) | 0.041 | 0.265 | **0.45**↓ | (±0.47) | 0.292 | 1.000 |
| AMD1: S-adenosylmethionine decarboxylase 1 | R82299 | 6q21-q22 | **0.59**↓ | (±0.28) | 0.054 | 0.265 | **0.80**↓ | (±0.50) | 0.410 | 1.000 |
| HIBADH: 3-hydroxyisobutyrate dehydrogenase | N77326 | 7p15 | **0.66**↓ | (±0.13) | 0.560 | 0.654 | 1.06 | (±0.37) | 0.820 | 1.000 |
| CYP3A4: cytochrome P450, subfamily IIIA (niphedipine oxidase), polypeptide 4 | R91078 | 7q21.1 | **0.70**↓ | (±0.05) | 0.372 | 0.479 | 0.88 | (±0.18) | 0.520 | 1.000 |
| PTDSS1: phosphatidylserine synthase 1 | H28984 | 8q22 | **0.65**↓ | (±0.16) | 0.175 | 0.287 | 1.02 | (±0.43) | 0.798 | 1.000 |
| CA2: carbonic anhydrase II | H23187 | 8q22 | **0.75**↓ | (±0.07) | 0.087 | 0.265 | 1.07 | (±0.09) | 0.351 | 1.000 |
| CYP2E: cytochrome P450, subfamily IIE (ethanol-inducible) | H50500 | 10q24.3-qter | **0.58**↓ | (±0.31) | 0.509 | 0.607 | 0.96 | (±0.60) | 0.991 | 1.000 |
| AMPD3: adenosine monophosphate deaminase (isoform E) | R01732 | 11p15 | 3.18 | (±1.93) | 0.442 | 0.548 | **0.75**↓ | (±0.35) | 0.540 | 1.000 |
| SIAT4C: sialyltransferase 4C (beta-galactosidase alpha-2,3-sialytransferase) | AA453898 | 11q23-q24 | **0.68**↓ | (±0.05) | 0.020 | 0.265 | 0.91 | (±0.13) | 0.360 | 1.000 |
| ALDH6A1: Aldehyde dehydrogenase 6 family, member A1 | N62179 | 14q24 | 0.94 | (±0.34) | 0.913 | 0.942 | **1.26**↑ | (±0.16) | 0.429 | 1.000 |
| DIO2: deiodinase, iodothyronine, type II | R62242 | 14q24.2-q24.3 | **0.42**↓ | (±0.38) | 0.219 | 0.327 | 0.99 | (±0.76) | 0.538 | 1.000 |
| GRP58: Glucose regulated protein, 58kD, | R33030 | 15q15 | **0.56**↓  **0.60**↓ | (±0.12) | 0.069 | 0.265 | 1.03 | (±0.47) | 0.958 | 1.000 |
| CYP19A1: cytochrome P450, family 19, subfamily A, polypeptide 1 | R32428 | 15q21.1 | **0.75**↓ | (±0.08) | 0.744 | 0.812 | 1.09 | (±0.14) | 0.923 | 1.000 |
| MPI: mannose phosphate isomerase | AA482198 | 15q22-qter | **0.62**↓ | (±0.14) | 0.191 | 0.300 | 1.15 | (±0.38) | 0.601 | 1.000 |
| CA11: Carbonic anhydrase XI | N52089 | 19q13.3 | **0.50**↓ | (±0.27) | 0.282 | 0.569 | 1.08 | (±0.88) | 0.533 | 1.000 |
| DPM1: dolichyl-phosphate mannosyltransferase polypeptide 1, catalytic subunit | AA004759 | 20q13.13 | **0.82**↓ | (±0.14) | 0.554 | 0.649 | 1.28 | (±0.31) | 0.194 | 1.000 |
| AHCY: S-adenosylhomocysteine hydrolase | AA485626 | 20cen-q13.1 | **0.65**↓ | (±0.23) | 0.081 | 0.265 | **1.30**↑ | (±0.42) | 0.210 | 1.000 |
| FTCD: formiminotransferase cyclodeaminase | W00987 | 21q22.3 | **0.76**↓ | (±0.20) | 0.724 | 0.795 | 1.23 | (±0.35) | 0.709 | 1.000 |
| HMOX1: heme oxygenase (decycling) 1 | T71757 | 22q12 | **3.75**↑ | (±2.58) | 0.693 | 0.769 | **0.70**↓ | (±0.54) | 0.491 | 1.000 |
| ALAS2: aminolevulinate, delta-, synthase 2 (sideroblastic/hypochromic anemia) | AA410346 | Xp11.21 | **0.48**↓ | (±0.15) | 0.010 | 0.225 | **0.80**↓ | (±0.41) | 0.299 | 1.000 |

| B: Generation of precursor metabolites and energy  GO:0006091 |  |  |  | | | |  | | | |
| --- | --- | --- | --- | --- | --- | --- | --- | --- | --- | --- |
| Gene name | GenBank no | chromosomal location | TGFβ − treated vs. control | | | | IMR-  treated vs. control | | | |
|  |  |  | ratio | SD | p-value | FDR | ratio | SD | p-value | FDR |
| SDHB: succinate dehydrogenase complex, subunit B, iron sulfur (Ip) | AA463565 | 1p36.1-p35 | **0.43**↓ | (±0.20) | 0.041 | 0.265 | **0.80**↓ | (±0.38) | 0.570 | 1.000 |
| LDHA: Lactate dehydrogenase A | AA489611 | 11p15.4 | 1.04 | (±0.43) | 0.839 | 0.885 | **1.55**↑ | (±0.29) | 0.430 | 1.000 |
| CKMT1B: creatine kinase, mitochondrial 1B | AA019482 | 15q15 | 1.05 | (±0.47) | 0.821 | 0.871 | **1.29**↑ | (±0.27) | 0.426 | 1.000 |
| PHKB: phosphorylase kinase, beta | AA476263 | 16q12-q13 | **0.58**↓ | (±0.04) | 0.067 | 0.265 | 0.85 | (±0.27) | 0.695 | 1.000 |
| UQCR: ubiquinol-cytochrome c reductase (6.4kD) subunit | R46837 | 19p13.3 | **0.42**↓ | (±0.40 | 0.123 | 0.265 | 0.90 | (±0.49) | 0.629 | 1.000 |
| HCCS: holocytochrome c synthase (cytochrome c heme-lyase) | AA281548 | Xp22.3 | **0.79**↓ | (±0.22) | 0.275 | 0.381 | 1.04 | (±0.24) | 0.963 | 1.000 |

| C: Nucleic acid binding  GO:0003676 |  |  |  | | | |  | | | |
| --- | --- | --- | --- | --- | --- | --- | --- | --- | --- | --- |
| Gene name | GenBank no | chromosomal location | TGFβ−treated vs. control | | | | IMR-treated vs. control | | | |
|  |  |  | ratio | SD | p-value | FDR | ratio | SD | p-value | FDR |
| PSEN2: presenilin 2 (Alzheimer disease 4) | AA152294 | 1q31-q42 | **0.76**↓ | (±0.18) | 0.518 | 0.616 | 1.06 | (±0.29) | 0.919 | 1.000 |
| BAP1: BRCA1 associated protein-1 (ubiquitin carboxy-terminal hydrolase) | H09065 | 3p21.31-p21.2 | **0.68**↓  **0.72**↓ | (±0.17)  (±0.19) | 0.236  0.447 | 0.341  0.553 | 1.08  1.07 | (±0.16)  (±0.19) | 0.621  0.755 | 1.000  1.000 |
| RARRES1: retinoic acid receptor responder (tazarotene induced) 1 | N94424 | 3q25.31 | **0.60**↓ | (±0.15) | 0.270 | 0.376 | 0.94 | (±0.15) | 0.738 | 1.000 |
| HIST1H2AC: histone 1, H2ac | AA453105 | 6p21.3 | **0.78**↓ | 0.24 | 0.369 | 0.477 | **0.78**↓ | 0.28 | 0.180 | 1.000 |
| MYB: v-myb myeloblastosis viral oncogene homolog (avian) | W86100 | 6q22-q23 | **0.73**↓ | (±0.18) | 0.141 | 0.269 | 0.95 | (±0.28) | 0.562 | 1.000 |
| PDAP1: PDGFA associated protein 1 | AA490300 | 7q11.21 | **0.62**↓ | (±0.24) | 0.260 | 0.365 | 1.39 | (±0.82) | 0.391 | 1.000 |
| CDK6: cyclin-dependent kinase 6 | H73724 | 7q21-q22 | **2.57**↑ | (±1.34) | 0.418 | 0.525 | **0.61**↓ | (±0.34) | 0.442 | 1.000 |
| CKS2: CDC28 protein kinase regulatory subunit 2 | AA397813 | 9q22 | **0.76**↓ | (±0.16) | 0.046 | 0.265 | 0.94 | (±0.13) | 0.504 | 1.000 |
| HELLS: helicase, lymphoid-specific | W25169 | 10q23-q24 | **0.64**↓ | (±0.21) | 0.495 | 0.595 | 1.05 | (±0.17) | 0.980 | 1.000 |
| TAB182: tankyrase 1-binding protein of 182 kDa | R74078 | 11q12.2 | **0.57**↓ | (±0.22) | 0.103 | 0.265 | 0.93 | (±0.32) | 0.891 | 1.000 |
| APC7: anaphase-promoting complex subunit 7 | T67474 | 12q13.12 | **0.77**↓ | (±0.07) | 0.141 | 0.269 | **0.80**↓ | (±0.13) | 0.221 | 1.000 |
| CDK2AP1: CDK2-associated protein 1 | R78607 | 12q24.31 | **0.65**↓ | (±0.10) | 0.133 | 0.269 | 1.03 | (±0.46) | 0.806 | 1.000 |
| PSEN1: presenilin 1 (Alzheimer disease 3) | AA403083 | 14q24.3 | **0.60**↓ | (±0.26) | 0.147 | 0.272 | 0.94 | (±0.41) | 0.567 | 1.000 |
| CDC25B: cell division cycle 25B | AA448755 | 20p13 | **0.59**↓ | (±0.28) | 0.216 | 0.232 | 0.99 | (±0.62) | 0.514 | 1.000 |
| LASS4: LAG1 longevity assurance homolog 4 (S. cerevisiae) | AA025779 | 19p13.2 | **0.62**↓ | 0.29 | 0.282 | 0.388 | **0.73**↓ | 0.33 | 0.339 | 1.000 |
| XRCC1: X-ray repair complementing defective repair in Chinese hamster cells 1 | AA425139 | 19q13.2 | 1.15 | (±0.43) | 0.914 | 0.943 | **0.70**↓ | (±0.21) | 0.301 | 1.000 |
| NXP-2: nuclear matrix protein NXP-2 | N73634 | 21q22.13 | **0.63**↓ | (±0.34) | 0.391 | 0.498 | 1.20 | (±0.67) | 0.894 | 1.000 |
| RBM10: RNA binding motif protein 10 | T68202 | Xp11.3 | **0.77**↓ | (±0.18) | 0.659 | 0.740 | **1.33**↑ | (±0.41) | 0.438 | 1.000 |
| SEPT6: septin 6 | R76772 | Xq24 | **0.55**↓ | (±0.23) | 0.437 | 0.543 | 1.01 | (±0.83) | 0.482 | 1.000 |
| SMARCA1: SWI/SNF related, matrix associated, actin dependent regulator of chromatin, subfamily a, member 1 | AA496809 | Xq25 | **0.69**↓ | (±0.23) | 0.101 | 0.115 | 1.01 | (±0.83) | 0.773 | 1.000 |
| MTCP1: mature T-cell proliferation 1 | AA029842 | Xq28 | **0.78**↓ | (±0.18) | 0.560 | 0.654 | 1.30 | (±0.53) | 0.531 | 1.000 |

| D: Transcription regulator activity  GO:0030528 |  |  |  | | | |  | | | |
| --- | --- | --- | --- | --- | --- | --- | --- | --- | --- | --- |
| Gene name | GenBank no | chromosomal location | TGFβ−treated vs. control | | | | IMR-treated vs. control | | | |
|  |  |  | ratio | SD | p-value | FDR | ratio | SD | p-value | FDR |
| CSDE1: cold shock domain containing E1, RNA-binding | AA504682 | 1p22 | **0.49**↓ | (±0.24) | 0.384 | 0.492 | 1.18 | (±0.79) | 0.756 | 1.000 |
| RLF: rearranged L-myc fusion sequence | R26070 | 1p32 | **0.54**↓ | (±0.27) | 0.467 | 0.571 | **0.77**↓ | (±0.29) | 0.540 | 1.000 |
| MTF1: metal-regulatory transcription factor 1 | R96603 | 1p33 | **0.81**↓ | (±0.13) | 0.605 | 0.694 | 1.13 | (±0.17) | 0.638 | 1.000 |
| ADAR: adenosine deaminase, RNA-specific | AA600189 | 1q21.1-q21.2 | **0.60**↓ | (±0.12) | 0.021 | 0.265 | 0.92 | (±0.20) | 0.823 | 1.000 |
| EPRS: glutamyl-prolyl-tRNA synthetase | AA599158 | 1q41-q42 | **0.80**↓ | (±0.19) | 0.314 | 0.420 | 0.99 | (±0.17) | 0.925 | 1.000 |
| RBM34: RNA binding motif protein 34 | AA448533 | 1q42.1-q43 | **0.75**↓ | (±0.18) | 0.442 | 0.548 | 1.02 | (±0.14) | 0.962 | 1.000 |
| DDX18: DEAD/H (Asp-Glu-Ala-Asp/His) box polypeptide 18 | R08935 | 2q21.2 | **0.66**↓ | (±0.31) | 0.610 | 0.698 | 1.10 | (±0.37) | 0.921 | 1.000 |
| EIF4E2: eukaryotic translation initiation factor 4E member 2 | W01534 | 2q37 | **3.54**↑ | (±1.90) | 0.390 | 0.495 | 0.90 | (±0.71) | 0.499 | 1.000 |
| LRRFIP1: leucine rich repeat (in FLII) interacting protein 1 | AA085597 | 2q37.3 | **0.71**↓ | (±0.20) | 0.265 | 0.370 | 0.97 | (±0.22) | 0.968 | 1.000 |
| RBM5: RNA binding motif protein 5 | W73892 | 3p21.3 | **0.68**↓ | (±0.15) | 0.045 | 0.265 | 1.13 | (±0.43) | 0.680 | 1.000 |
| ZFP: zinc finger protein | H53499 | 3p22.3-p21.1 | **0.69**↓ | (±0.11) | 0.160 | 0.277 | 0.88 | (±0.06) | 0.491 | 1.000 |
| RPL32: ribosomal protein L32 | R43544 | 3p25-p24 | 0.87 | (±0.19) | 0.251 | 0.356 | **1.27**↑ | (±0.13) | 0.243 | 1.000 |
| TFDP2: transcription factor Dp-2 | AA465444 | 3q23 | **0.57**↓ | (±0.18) | 0.036 | 0.265 | 0.97 | (±0.33) | 0.920 | 1.000 |
| MSX1: Msh homeo box homolog 1 (Drosophila) | R33154 | 4p16.3-p16.1 | **0.54**↓ | (±0.24) | 0.199 | 0.308 | 1.00 | (±0.75) | 0.489 | 1.000 |
| TCF7: transcription factor 7 (T-cell specific, HMG-box) | AA480071 | 5q31.1 | **0.76**↓ | (±0.49) | 0.180 | 0.291 | **0.80**↓ | (±0.42) | 0.297 | 1.000 |
| HNRPH1: heterogeneous nuclear ribonucleoprotein H1 | W96114 | 5q35.3 | **0.53**↓ | (±0.24) | 0.572 | 0.665 | 0.98 | (±0.39) | 0.739 | 1.000 |
| ZNF193: zinc finger protein 193 | AA252169 | 6p21.3 | **0.49**↓ | (±0.20) | 0.102 | 0.265 | 0.96 | (±0.68) | 0.482 | 1.000 |
| HSF2: heat shock transcription factor 2 | AA250730 | 6pter-p25.1 | **0.68**↓ | (±0.15) | 0.475 | 0.578 | 1.02 | (±0.35) | 0.766 | 1.000 |
| AEBP1: AE binding protein 1 | AA490462  AA490684 | 7p13 | **0.48**↓  **0.60**↓ | (±0.34)  (±0.32) | 0.484  0.487 | 0.587  0.597 | 0.99  1.40 | (±0.50)  (±1.15) | 0.663  0.781 | 1.000  1.000 |
| P37NB: 37 kDa leucine-rich repeat (LRR) protein | AA423944 | 7q11.22 | **0.72**↓ | (±0.08) | 0.478 | 0.581 | 1.23 | (±0.46) | 0.692 | 1.000 |
| TCF8: transcription factor 8 (represses interleukin 2 expression) | R22087 | 10p11.2 | **0.62**↓ | (±0.38) | 0.371 | 0.479 | **0.74**↓ | (±0.39) | 0.392 | 1.000 |
| PRP18: pre-mRNA processing factor 18 | H82325 | 10p12.33 | **0.52**↓ | (±0.28) | 0.086 | 0.265 | 0.97 | (±0.22) | 0.791 | 1.000 |
| E2F8: E2F transcription factor 8 | W03979 | 11p15.1 | **3.43**↑ | (±1.94) | 0.485 | 0.587 | 0.92 | (±0.88) | 0.508 | 1.000 |
| CSRP3: cysteine and glycine-rich protein 3 | AA195959 | 11p15.1 | **0.53**↓ | (±0.03) | 0.000 | 0.115 | 0.90 | (±0.46) | 0.652 | 1.000 |
| RELA: v-rel reticuloendotheliosis viral oncogene homolog A | AA443546 | 11q13 | **0.55**↓ | (±0.10) | 0.078 | 0.265 | 1.03 | (±0.55) | 0.743 | 1.000 |
| DRAP1: DR1-associated protein 1 | AA421977  AA406285 | 11q13.3 | **0.53**↓  **0.60**↓ | (±0.22)  (±0.19) | 0.111 | 0.265 | 0.99  1.11 | (±0.29)  (±0.26) | 0.757 | 1.000 |
| ATF7: activating transcription factor 7 | W45531 | 12q13 | **0.70**↓ | (±0.06) | 0.514 | 0.612 | 1.04 | (±0.09) | 0.939 | 1.000 |
| PFDN5: prefoldin 5 | AA446453 | 12q13.13 | **0.53**↓ | (±0.13) | 0.020 | 0.265 | 1.03 | (±0.33) | 0.953 | 1.000 |
| TFDP1: transcription factor Dp-1 | W33012 | 13q34 | **0.76**↓ | (±0.009) | 0.858 | 0.898 | 0.99 | (±0.26) | 0.957 | 1.000 |
| JDP2: jun dimerization protein 2 | AA443659 | 14q24.3 | **0.65**↓ | (±0.08) | 0.570 | 0.663 | 1.13 | (±0.44) | 0.773 | 1.000 |
| EIF2B2: eukaryotic translation initiation factor 2B, subunit 2 (beta, 39kD) | R86304 | 14q24.3 | **0.64**↓ | (±0.19) | 0.018 | 0.261 | 1.05 | (±0.25) | 0.765 | 1.000 |
| PIG7: LPS-induced TNF-alpha factor | AA625666 | 16p13.3-p12 | 0.80 | (±0.30) | 0.584 | 0.643 | **1.30**↑ | (±0.29) | 0.338 | 1.000 |
| TFAP4 Transcription factor AP-4 (activating enhancer binding protein 4 | AA284693 | 16p13 | **0.56**↓ | (±0.15) | 0.090 | 0.265 | 1.08 | (±0.37) | 0.766 | 1.000 |
| RNPS1: RNA binding protein S1, serine-rich domain | AA496837 | 16p13.3 | **0.65**↓ | (±0.17) | 0.120 | 0.265 | 1.03 | (±0.03) | 0.942 | 1.000 |
| DDX19: DEAD/H (Asp-Glu-Ala-Asp/His) box polypeptide 19 | R64251 | 16q 22 | **0.46**↓ | (±0.18) | 0.230 | 0.335 | 1.18 | (±0.94) | 0.580 | 1.000 |
| ROK1: ATP-dependent RNA helicase | W73792 | 17q21.1 | **0.41**↓ | (±0.25) | 0.337 | 0.443 | 0.81 | (±0.47) | 0.406 | 1.000 |
| SFRS1: splicing factor, arginine/serine-rich 1 (splicing factor 2, alternate splicing factor) | T65902 | 17q21.3-q22 | **0.47**↓ | (±0.16) | 0.181 | 0.292 | 1.12 | (±0.59) | 0.921 | 1.000 |
| ZNF161: zinc finger protein 161 | AA232647 | 17q23.3 | **0.71**↓  **0.75**↓ | (±0.11)  (±0.13) | 0.339 | 0.445 | 0.94  1.08 | (±0.31)  (±0.11) | 0.608 | 1.000  1.000 |
| ZNF24: zinc finger protein 24 (KOX 17) | AA447098 | 18q12 | **0.73**↓ | (±0.07) | 0.651 | 0.732 | 1.10 | (±0.46) | 0.712 | 1.000 |
| TLE1: transducin-like enhancer of split 1 (E(sp1) homolog, Drosophila) | T61445 | 19p13.3 | **0.64**↓ | (±0.06) | 0.023 | 0.265 | 0.90 | (±0.20) | 0.242 | 1.000 |
| ZNF358: zinc finger protein 358 | H20045 | 19p13 | **0.64**↓ | (±0.09) | 0.304 | 0.411 | 0.93 | (±0.30) | 0.621 | 1.000 |
| MRPL4: mitochondrial ribosomal protein L4 | AA490981 | 19p13.2 | **0.68**↓ | (±0.10) | 0.103 | 0.265 | 0.98 | (±0.14) | 0.743 | 1.000 |
| RPS5: ribosomal protein S5 | AA456616 | 19q13.4 | **0.66**↓ | (±0.13) | 0.030 | 0.265 | 0.90 | (±0.16) | 0.503 | 1.000 |
| NUCB1: nucleobindin 1 | AA452725 | 19q13.2-q13.4 | **0.64**↓ | (±0.11) | 0.337 | 0.444 | 0.95 | (±0.20) | 0.701 | 1.000 |
| EZF-2: endothelial zinc finger protein 2 | R63318 | 19q13.43 | **0.58**↓ | (±0.22) | 0.061 | 0.265 | 0.87 | (±0.23) | 0.374 | 1.000 |
| ID1: inhibitor of DNA binding 1, dominant negative helix-loop-helix protein | AA457158 | 20q11 | **0.74**↓ | (±0.18) | 0.210 | 0.317 | 1.06 | (±0.13) | 0.807 | 1.000 |
| TCEA2: transcription elongation factor A (SII), 2 | AA412500 | 20q13.33 | **0.61**↓ | (±0.36) | 0.241 | 0.345 | **0.72**↓ | (±0.33) | 0.161 | 1.000 |
| SF3A1 splicing factor 3a, subunit 1. 120kD | T72698 | 22q12.2 | **0.71**↓ | (±0.13) | 0.510 | 0.609 | 1.11 | (±0.36) | 0.967 | 1.000 |

| E: Signal transducer activity GO:0004871,  Signal transduction GO:0007165 and  Cell communication GO:0007154 |  |  |  | | | |  | | | |
| --- | --- | --- | --- | --- | --- | --- | --- | --- | --- | --- |
| Gene name | GenBank no | chromosomal location | TGFβ−treated vs. control | | | | IMR-treated vs. control | | | |
|  |  |  | ratio | SD | p-value | FDR | ratio | SD | p-value | FDR |
| EPS15: epidermal growth factor receptor pathway substrate 15 | AA490223 | 1p32 | **0.69**↓ | (±0.26) | 0.432 | 0.539 | 1.40 | (±0.89) | 0.762 | 1.000 |
| TIE1: tyrosine kinase with immunoglobulin-like and EGF-like domains 1 | AA432062 | 1p34-p33 | **0.68**↓ | 0.20 | 0.084 | 0.265 | **0.72**↓ | 0.13 | 0.070 | 1.000 |
| GRIK3: Glutamate receptor, ionotropic, kainate 3 | AA058857 | 1p34-p33 | **0.36**↓ | (±0.10) | 0.094 | 0.265 | 0.94 | (0.49)± | 0.916 | 1.000 |
| CTNNBIP1: catenin, beta interacting protein 1 | R78539 | 1p36.22 | **0.59**↓ | (±0.33) | 0.205 | 0.313 | 1.23 | (±0.91) | 0.836 | 1.000 |
| CRABP2: cellular retinoic acid binding protein 2 | AA598508 | 1q21.3 | **0.72**↓ | (±0.20) | 0.451 | 0.556 | 1.06 | (±0.19) | 0.870 | 1.000 |
| RIT1: Ras-like without CAAX 1 | AA027840 | 1q22 | **0.74**↓ | (±0.19) | 0.530 | 0.628 | 1.04 | (±0.22) | 0.905 | 1.000 |
| GPA33: glycoprotein A33 (transmembrane) | AA055862 | 1q24.1 | **0.72**↓ | (±0.10) | 0.679 | 0.756 | **0.80**↓ | (±0.16) | 0.752 | 1.000 |
| MAPKAPK2: mitogen-activated protein kinase-activated protein kinase 2 | AA455056 | 1q32 | **0.61**↓ | (±0.10) | 0.031 | 0.265 | **0.80**↓ | (±0.21) | 0.385 | 1.000 |
| ARHE: ras homolog gene family, member E | AA443302 | 2q23.3 | **0.63**↓ | (±0.21) | 0.080 | 0.265 | 1.00 | (±0.33) | 0.928 | 1.000 |
| DGKD: diacylglycerol kinase, delta (130kD) | AA280691 | 2q37.1 | **0.60**↓ | (±0.00) | 0.375 | 0.483 | 1.18 | (±0.72) | 0.819 | 1.000 |
| CTNNB1: catenin (cadherin-associated protein), beta 1, 88kDa | AA442092 | 3p21 | **0.65**↓ | (±0.06) | 0.628 | 0.712 | 0.99 | (±0.08) | 0.948 | 1.000 |
| CTDSPL: CTD (carboxy-terminal domain, RNA polymerase II, polypeptide A) small phosphatase-like | R01638 | 3p21.3 | **0.71**↓ | (±0.23) | 0.281 | 0.387 | 1.04 | (±0.05) | 0.784 | 1.000 |
| WNT5A: wingless-type MMTV integration site family, member 5A | W49672 | 3p21-p14 | **0.72**↓ | (±0.19) | 0.307 | 0.414 | 1.07 | (±0.16) | 0.899 | 1.000 |
| TGFBR2: transforming growth factor, beta receptor II (70-80kD) | AA487034 | 3p22 | **0.71**↓ | (±0.17) | 0.450 | 0.555 | 1.06 | (±0.14) | 0.906 | 1.000 |
| TM4SF1: transmembrane 4 superfamily member 1 | AA487893 | 3q21-q25 | **0.66**↓ | (±0.14) | 0.259 | 0.364 | 0.95 | (±0.05) | 0.803 | 1.000 |
| SIAH2: seven in absentia homolog 2 (Drosophila) | AA029041 | 3q25 | **0.65**↓ | (±0.13) | 0.037 | 0.265 | 1.01 | (±0.19) | 0.948 | 1.000 |
| CENTB2: centaurin, beta 2 | AA490493 | 3q29 | **0.63**↓ | (±0.14) | 0.659 | 0.740 | 1.07 | (±0.13) | 0.997 | 1.000 |
| GYPE: glycophorin E | N48137 | 4q28-q31 | **0.49**↓ | (±0.16) | 0.119 | 0.265 | 1.06 | (±0.69) | 0.709 | 1.000 |
| GPM6A: glycoprotein M6A | AA448033 | 4q34 | **0.67**↓ | (±0.21) | 0.632 | 0.715 | 1.06 | (±0.46) | 0.728 | 1.000 |
| PDZD2: PDZ domain containing 2 | AA405458 | 5p13.2 | **0.62**↓ | (±0.07) | 0.049 | 0.265 | **0.83**↓ | (±0.12) | 0.350 | 1.000 |
| PIK3R1: phosphoinositide-3-kinase, regulatory subunit, polypeptide 1 (p85 alpha) | R54050 | 5q12-q13 | **0.61**↓ | (±0.11) | 0.539 | 0.636 | 1.25 | (±0.75) | 0.832 | 1.000 |
| IQGAP2 IQ motif containing GTPase activating protein 2 | W32272 | 5q13.2 | **0.56**↓ | (±0.09) | 0.050 | 0.265 | 1.04 | (±0.48) | 0.955 | 1.000 |
| PRL:prolactin | AA133920 | 6p22.2-p21.3 | **0.66**↓ | 0.31 | 0.368 | 0.470 | **0.69**↓ | 0.29 | 0.375 | 1.000 |
| SSR1: signal sequence receptor, alpha (translocon-associated protein alpha) | AA099394 | 6p24.3 | **0.75**↓ | (±0.08) | 0.693 | 0.769 | 1.05 | (±0.22) | 0.831 | 1.000 |
| TRDN: triadin | AA404293 | 6q22-q23 | **0.76**↓ | (±0.12) | 0.598 | 0.688 | 1.10 | (±0.16) | 0.793 | 1.000 |
| SGK: serum/glucocorticoid regulated kinase | AA486082 | 6q23 | **0.79**↓ | (±0.21) | 0.350 | 0.457 | 0.96 | (±0.25) | 0.719 | 1.000 |
| AKAP12:A kinase (PRKA) anchor protein (gravin) 12 | AA478542 | 6q24-q25 | **0.75**↓ | 0.41 | 0.246 | 0.351 | **0.77**↓ | 0.35 | 0.256 | 1.000 |
| EGFR: epidermal growth factor receptor (erythroblastic leukemia viral (v-erb-b) oncogene homolog, avian) | R35665  W48713 | 7p12 | **0.47**↓  **0.75**↓ | (±0.04)  (±0.13) | 0.022  0.444 | 0.265  0.550 | 1.02  1.09 | (±0.63)  (±0.10) | 0.806  0.693 | 1.000  1.000 |
| EPHA1: ephrin receptor EphA1 | N90246 | 7q34 | **0.66**↓  **0.63**↓ | (±0.10)  (±0.07) | 0.066 | 0.265 | 1.27  1.00 | (±0.66) (±0.15)) | 0.658 | 1.000 |
| PKIA: protein kinase (cAMP-dependent, catalytic) inhibitor alpha | AA281667 | 8q21.11 | **0.73**↓ | (±0.09) | 0.705 | 0.778 | 1.01 | (±0.27) | 0.954 | 1.000 |
| TIEG: TGFB inducible early growth response | R79935 | 8q22.2 | **0.71**↓ | (±0.13) | 0.016 | 0.257 | 0.88 | (±0.08) | 0.214 | 1.000 |
| PPP6C: protein phosphatase 6, catalytic subunit | AA521083 | 9q33.3 | **0.77**↓ | (±0.19) | 0.364 | 0.427 | 0.97 | (±0.22) | 0.761 | 1.000 |
| STAM: signal transducing adaptor molecule (SH3 domain and ITAM motif) 1 | AA485996 | 10p14-p13 | **0.56**↓ | (±0.26) | 0.186 | 0.297 | **0.82**↓ | (±0.16) | 0.382 | 1.000 |
| CARP: cardiac ankyrin repeat protein,cytokine inducible nuclear protein C193 | AA488072 | 10q23.31 | **0.77**↓ | (±0.16) | 0.038 | 0.265 | **0.82**↓ | (±0.17) | 0.073 | 1.000 |
| RNTRE: related to the N terminus of tre | AA281057  AA281137 | 10p13 | **0.78**↓  **0.71**↓ | (±0.25)  (±0.21) | 0.691  0.651 | 0.767  0.732 | 1.04  1.14 | (±0.63)  (±0.12) | 0.866  0.688 | 1.000  1.000 |
| BDNF: brain-derived neurotrophic factor | AA262988 | 11p13 | **0.60**↓ | (±0.15) | 0.274 | 0.380 | 1.01 | (±0.29) | 0.833 | 1.000 |
| GPR48: G protein-coupled receptor 48 | H77855 | 11p14-p13 | **0.64**↓ | (±0.11) | 0.380 | 0.488 | 0.96 | (±0.25) | 0.959 | 1.000 |
| PTH: parathyroid hormone | W37306 | 11p15.3-p15.1 | **0.55**↓ | (±0.28) | 0.190 | 0.300 | 1.11 | (±1.00) | 0.608 | 1.000 |
| IGF2: insulin-like growth factor 2 (somatomedin A) | N54596 | 11p15.5 | **0.57**↓  **0.63**↓ | (±0.16)  (±0.17) | 0.241  0.335 | 0.345  0.441 | 0.92  1.23 | (±0.27)  (±0.86) | 0.565  0.912 | 1.000  1.000 |
| MS4A1: membrane-spanning 4-domains, subfamily A, member 1 (Fc fragment of IgE, high affinity I, receptor for; beta polypeptide | N91385 | 11q12-q13.1 | **1.24**↑ | (±0.66) | 0.612 | 0.699 | **1.63**↑ | (±0.62) | 0.323 | 1.000 |
| DRPLA: dentatorubral-pallidoluysian atrophy (atrophin-1) | H08642 | 12p13.31 | **0.65**↓ | (±0.42) | 0.330 | 0.436 | **0.80**↓ | (±0.53) | 0.364 | 1.000 |
| DGKA: diacylglycerol kinase, alpha (80kD) | AA456900 | 12q13.3 | **0.47**↓ | (±0.17) | 0.149 | 0.272 | 1.36 | (±1.40) | 0.810 | 1.000 |
| FLT1: fms-related tyrosine kinase 1 (vascular endothelial growth factor/vascular permeability factor receptor) | AA058828 | 13q12 | 2.20 | (±1.91) | 0.497 | 0.597 | **0.56**↓ | (±0.16) | 0.429 | 1.000 |
| AKAP11: A kinase (PRKA) anchor protein 11 | W00867 | 13q14.11 | **3.48**↑ | (±2.06) | 0.226 | 0.332 | 0.98 | (±0.44) | 0.657 | 1.000 |
| EFNB2: ephrin-B2 | AA461424  AA461108 | 13q33 | **0.79**↓  **0.77**↓ | (±0.20)  (±0.06) | 0.983  0.771 | 0.989  0.833 | 1.23  1.06 | (±0.13)  (±0.10) | 0.739  0.806 | 1.000  1.000 |
| ARHGEF7: Rho guanine nucleotide exchange factor (GEF) 7 | AA457036 | 13q34 | **0.58**↓ | (±0.25) | 0.300 | 0.407 | 1.15 | (±0.81) | 0.718 | 1.000 |
| CYFIP1: cytoplasmic FMR1 interacting protein 1 | AA598583 | 15q11 | **0.41**↓ | (±0.23) | 0.452 | 0.557 | 0.99 | (±0.42) | 0.726 | 1.000 |
| APBA2: amyloid beta (A4) precursor protein-binding, family A, member 2 (X11-like) | R55789 | 15q11-q12 | **0.58**↓ | (±0.32) | 0.309 | 0.415 | 0.85 | (±0.29) | 0.494 | 1.000 |
| MAPK6: mitogen-activated protein kinase 6 | H17504 | 15q21 | **0.55**↓ | (±0.45) | 0.470 | 0.574 | 1.18 | (±0.61) | 0.871 | 1.000 |
| NEDD4: neural precursor cell expressed, developmentally down-regulated 4 | AA442095 | 15q21.3 | **0.66**↓ | (±0.17) | 0.389 | 0.496 | 1.11 | (±0.11) | 0.682 | 1.000 |
| SIAH1: seven in absentia homolog 1 (Drosophila) | AA447531 | 16q12 | **0.57**↓ | (±0.23) | 0.055 | 0.265 | 0.97 | (±0.55) | 0.920 | 1.000 |
| PRKCB1: protein kinase C, beta 1 /cDNA DKFZp761J0720 | AA479102 | 16p11.2 | **1.70**↑ | (±0.75) | 0.392 | 0.499 | **0.69**↓ | (±0.19) | 0.376 | 1.000 |
| SH2B: SH2-B homolog | W23931 | 16p11.2 | **0.72**↓ | (±0.25) | 0.252 | 0.358 | 1.01 | (±0.09) | 0.951 | 1.000 |
| ABCC1: ATP-binding cassette, sub-family C (CFTR/MRP), member 1 | AA495766 | 16p13.12 | **0.65**↓ | (±0.15) | 0.022 | 0.265 | 1.00 | (±0.17) | 0.931 | 1.000 |
| MAP2K4: mitogen-activated protein kinase kinase 4 | AA293050 | 17p11.2 | **0.65**↓ | (±0.20) | 0.427 | 0.534 | 1.39 | (±0.89) | 0.873 | 1.000 |
| TIP-1: Tax interaction protein 1 | AA434504 | 17p13 | 1.04 | (±0.07) | 0.961 | 0.976 | **1.30**↑ | (±0.18) | 0.530 | 1.000 |
| ABR: Active BCR-related gene | W24076 | 17p13.3 | **0.59**↓ | (±0.08) | 0.343 | 0.450 | 1.27 | (±0.90) | 0.941 | 1.000 |
| EFNB3: ephrin-B3 | AA485795 | 17p13.1-p11.2 | **0.62**↓ | (±0.39) | 0.294 | 0.401 | 1.36 | (±0.91) | 0.846 | 1.000 |
| AKAP10: A kinase (PRKA) anchor protein 10 | R21506 | 17pter-qter | **0.56**↓ | (±0.24) | 0.444 | 0.550 | 1.18 | (±0.93) | 0.711 | 1.000 |
| NLK: nemo-like kinase | R70769 | 17q11.2 | **0.55**↓ | (±0.24) | 0.298 | 0.504 | 0.85 | (±0.39) | 0.499 | 1.000 |
| THRAP4: Thyroid hormone receptor associated protein 4 | N76581 | 17q21.1 | **3.79**↑ | (±2.45) | 0.491 | 0.592 | 1.02 | (±0.60) | 0.561 | 1.000 |
| PECAM1: platelet/endothelial cell adhesion molecule (CD31 antigen) | R22412 | 17q23 | **0.68**↓ | (±0.06) | 0.243 | 0.348 | 0.95 | (±0.18) | 0.644 | 1.000 |
| RPS6KB1: ribosomal protein S6 kinase, 70kD, polypeptide 1 | AA425446 | 17q23.1 | **0.55**↓ | (±0.30) | 0.123 | 0.265 | 0.99 | (±0.51) | 0.720 | 1.000 |
| RALBP1: ralA binding protein 1 | AA085990 | 18p11.3 | **0.67**↓ | (±0.14) | 0.122 | 0.265 | 1.28 | (±0.43) | 0.391 | 1.000 |
| DSC2: desmocollin 2 | AA074677 | 18q11.2 | **0.42**↓ | (±0.27) | 0.153 | 0.274 | 1.06 | (±0.73) | 0.597 | 1.000 |
| SMAD4: SMAD, mothers against DPP homolog 4 (Drosophila) | AA456439 | 18q21.1 | **0.78**↓ | (±0.14) | 0.186 | 0.296 | 1.00 | (±0.23) | 0.870 | 1.000 |
| MAST1: microtubule associated serine/threonine kinase 1 | AA479623 | 19p13.2 | **0.59**↓  **0.51**↓ | (±0.48)  (±0.18) | 0.146  0.274 | 0.272  0.380 | 1.00  0.71 | (±0.88)  (±0.31) | 0.575  0.209 | 1.000  1.000 |
| FPR1: formyl peptide receptor 1 | AA425249 | 19q13.4 | **0.44**↓ | (±0.25) | 0.018 | 0.261 | **0.79**↓ | (±0.37) | 0.371 | 1.000 |
| PLCG1: Phospholipase C, gamma 1 (formerly subtype 148) | R76365 | 20q12-q13.1 | **0.48**↓ | (±0.23) | 0.050 | 0.265 | **0.81**↓ | (±0.48) | 0.656 | 1.000 |
| ARHGAP8: Rho GTPase activating protein 8 | R69153 | 22q13.31 | **0.61**↓ | (±0.09) | 0.017 | 0.261 | **0.80**↓ | (±0.23) | 0.307 | 1.000 |
| MAPK8IP2: mitogen-activated protein kinase 8 interacting protein 2 | R22306 | 22q13.33 | **0.56**↓ | (±0.29) | 0.073 | 0.265 | 1.08 | (±0.71) | 0.849 | 1.000 |
| GRP64:G protein-coupled receptor 64 | H77479 | Xp22.13 | **3.92**↑ | (±2.23) | 0.364 | 0.472 | 1.09 | (±1.05 | 0.525 | 1.000 |
| EFNB1: ephrin-B1 | AA428778 | Xq12 | **5.14**↑ | (±2.92 | 0.231 | 0.336 | 1.09 | (±1.03) | 0.539 | 1.000 |
| IL13RA1: Alpha chain of the interleukin 13 receptor; binds interleukin-13 (IL13) with low affinity | AA411324 | Xq24 | **0.75**↓ | (±0.22) | 0.534 | 0.631 | 1.16 | (±0.37) | 0.906 | 1.000 |

| F: Cellular macromolecule metabolism, GO:0044260 |  |  |  | | | |  | | | |
| --- | --- | --- | --- | --- | --- | --- | --- | --- | --- | --- |
| Gene name | GenBank no | chromosomal location | TGFβ−treated vs. control | | | | IMR-treated vs. control | | | |
|  |  |  | ratio | SD | p-value | FDR | ratio | SD | p-value | FDR |
| DNAJB4: DnaJ (Hsp40) homolog, subfamily B, member 4 | AA084517 | 1p31.1 | 1.05 | (±0.43) | 0.766 | 0.830 | **1.56**↑ | (±0.23) | 0.328 | 1.000 |
| DDOST: dolichyl-diphosphooligosaccharide-protein glycosyltransferase | H96850 | 1p36.1 | **0.73**↓ | (±0.20) | 0.299 | 0.334 | 1.16 | (±0.28) | 0.328 | 1.000 |
| ECE1: endothelin converting enzyme 1 | AA279429 | 1p36.12 | 0.93 | (±0.36) | 0.542 | 0.638 | **1.21**↑ | (±0.16) | 0.659 | 1.000 |
| CTSS: cathepsin S | AA236164 | 1q21 | **0.53**↓ | (±0.04) | 0.327 | 0.433 | 0.90 | (±0.30) | 0.580 | 1.000 |
| ADAM15: a disintegrin and metalloproteinase domain 15 (metargidin) | AA292676 | 1q21.3 | **0.44**↓ | (±0.14) | 0.320 | 0.427 | 1.05 | (±0.70) | 0.572 | 1.000 |
| CTSE: cathepsin E (gastric aspartyl protease (acid protease)) | H94487 | 1q31 | **1.75**↑ | (±0.75) | 0.510 | 0.609 | 0.80 | (±0.38) | 0.505 | 1.000 |
| REN: renin | AA455535 | 1q32 | **0.44**↓ | (±0.32) | 0.133 | 0.269 | **0.88**↓ | (±0.62) | 0.385 | 1.000 |
| VRK2: vaccinia related kinase 2 | AA490617 | 2p16-p15 | 0.99 | (±0.16) | 0.925 | 0.951 | **0.78**↓ | (±0.22) | 0.853 | 1.000 |
| P5: protein disulfide isomerase-related protein | R01669 | 2p25.1 | **0.65**↓ | (±0.10) | 0.074 | 0.265 | 0.89 | (±0.27) | 0.412 | 1.000 |
| TFPI: Tissue factor pathway inhibitor (lipoprotein-associated coagulation inhibitor) | R66057 | 2q31-q32.1 | **0.50**↓ | (±0.08) | 0.315 | 0.421 | 1.25 | (±0.76) | 0.979 | 1.000 |
| PPP1R7 Protein phosphatase 1, regulatory subunit 7 | AA459572 | 2q37.3 | **0.60**↓ | (±0.35) | 0.102 | 0.265 | 0.97 | (±0.88) | 0.885 | 1.000 |
| LXN: latexin | W47077 | 3q25.32 | **0.57**↓ | (±0.36) | 0.347 | 0.454 | 0.97 | (±0.34) | 0.465 | 1.000 |
| PSMD2: proteasome (prosome, macropain) 26S subunit, non-ATPase, 2 | AA455193 | 3q27.1 | **0.72**↓ | 0.39 | 0.341 | 0.448 | **0.73**↓ | 0.50 | 0.362 | 1.000 |
| CPZ: carboxypeptidase Z | AA427724 | 4p16.1 | **0.80**↓ | (±0.16) | 0.318 | 0.425 | 1.17 | (±0.93) | 0.764 | 1.000 |
| BHMT2: betaine-homocysteine methyltransferase 2 | R98074 | 5q13 | 0.61 | (±0.31) | 0.222 | 0.329 | **0.85**↓ | (±0.33) | 0.480 | 1.000 |
| RNF5: ring finger protein 5 | AA402960 | 6p21.3 | **0.67**↓ | (±0.13) | 0.107 | 0.265 | 0.96 | (±0.18) | 0.630 | 1.000 |
| SERPINB6: serine (or cysteine) proteinase inhibitor, clade B (ovalbumin), member 6 | AA410517 | 6p25 | **0.72**↓ | (±0.13) | 0.369 | 0.477 | **0.74**↓ | (±0.27) | 0.254 | 1.000 |
| PRSS2: protease, serine, 2 (trypsin 2) | AA284528 | 7q34 | **0.68**↓ | (±0.19) | 0.117 | 0.265 | 1.05 | (±0.14) | 0.655 | 1.000 |
| PPIF: peptidylprolyl isomerase F (cyclophilin F) | H05580 | 10q22-q23 | **0.78**↓ | (±0.12) | 0.229 | 0.334 | 1.02 | (±0.13) | 0.897 | 1.000 |
| SPUVE: protease, serine, 23 | R76394 | 11q14 | **3.72**↑ | (±2.26) | 0.503 | 0.602 | 2.03 | (±2.15) | 0.589 | 1.000 |
| MMP7: matrix metalloproteinase 7 (matrilysin, uterine) | AA031513 | 11q21-q22 | **0.61**↓ | (±0.12) | 0.011 | 0.227 | 0.94 | (±0.19) | 0.601 | 1.000 |
| CASP4: caspase 4, apoptosis-related cysteine protease | H45000 | 11q22.2-q22.3 | **3.83**↑ | (±2.44) | 0.282 | 0.388 | 0.91 | (±0.58) | 0.537 | 1.000 |
| CRYAB:crystallin, alpha B | AA504943 | 11q22.3-q23.1 | **0.50**↓ | (±0.08) | 0.096 | 0.265 | **0.72**↓ | (±0.30) | 0.219 | 1.000 |
| ST14: suppression of tumorigenicity 14 (colon carcinoma, matriptase, epithin) | AA489246 | 11q24-q25 | **0.67**↓ | (±0.36) | 0.188 | 0.298 | 0.94 | (±0.36) | 0.821 | 1.000 |
| UBE2N: ubiquitin-conjugating enzyme E2N (UBC13 homolog, yeast) | AA490124 | 12q21.33 | **0.76**↓  **0.79**↓ | (±0.16)  (±0.17) | 0.294  0.558 | 0.401  0.652 | 1.25  1.12 | (±0.26)  (±0.19) | 0.281  0.684 | 1.000  1.000 |
| PSMA3: proteasome (prosome, macropain) subunit, alpha type, 3 | AA465593 | 14q23 | **0.58**↓ | (±0.19 | 0.319 | 0.426 | 1.11 | (±0.68) | 0.700 | 1.000 |
| CTSH: cathepsin H; lysosomal cysteine (thiol) proteinase | AA487346 | 15q24-q25 | **0.74**↓ | (±0.15) | 0.115 | 0.265 | 1.09 | (±0.21) | 0.603 | 1.000 |
| UBE2I: ubiquitin-conjugating enzyme E2I (UBC9 homolog, yeast) | AA487197 | 16p13.3 | 1.19 | (±0.34) | 0.489 | 0.591 | **1.43**↑ | (±0.25) | 0.154 | 1.000 |
| PSMB6: proteasome (prosome, macropain) subunit, beta type, 6 | AA070997 | 17p13 | **0.76**↓ | (±0.16) | 0.093 | 0.265 | 1.00 | (±0.12) | 0.972 | 1.000 |
| DUSP14: dual specificity phosphatase 14 | AA129677 | 17q12 | **0.51**↓ | (±0.12) | 0.161 | 0.277 | 0.94 | (±0.22) | 0.955 | 1.000 |
| NMT1: N-myristoyltransferase 1 | AA448910 | 17q21.31 | **0.40**↓ | (±0.30) | 0.067 | 0.265 | 1.11 | (±0.92) | 0.760 | 1.000 |
| SPINT2: serine protease inhibitor, Kunitz type, 2 | AA459039 | 19q13.1 | **0.79**↓ | (±0.15) | 0.537 | 0.634 | 1.14 | (±0.12) | 0.524 | 1.000 |
| KLK6: kallikrein 6 (neurosin, zyme) | AA454743 | 19q13.3 | **0.62**↓ | (±0.08) | 0.037 | 0.265 | 0.96 | (±0.26) | 0.739 | 1.000 |
| CST3: cystatin C (amyloid angiopathy and cerebral hemorrhage) | AA599177 | 20p11.21 | **0.43**↓ | (±0.35) | 0.318 | 0.425 | 0.93 | (±0.62) | 0.491 | 1.000 |
| TGM2: transglutaminase 2 (C polypeptide, protein-glutamine-gamma-glutamyltransferase) | R97066 | 20q12 | **0.62**↓ | (±0.30) | 0.171 | 0.284 | **0.73**↓ | (±0.24) | 0.182 | 1.000 |
| PFDN4: prefoldin 4 | AA253430 | 20q13 | 1.08 | (±0.65) | 0.807 | 0.859 | **1.46**↑ | (±0.67) | 0.344 | 1.000 |

| G: Inflammatory response  GO:0006954 |  |  |  | | | |  | | | |
| --- | --- | --- | --- | --- | --- | --- | --- | --- | --- | --- |
| Gene name | GenBank no | chromosomal location | TGFβ−treated vs. control | | | | IMR-treated vs. control | | | |
|  |  |  | ratio | SD | p-value | FDR | ratio | SD | p-value | FDR |
| CSF3R: colony stimulating factor 3 receptor (granulocyte) | AA443000 | 1p35-p34.3 | **0.77**↓ | (±0.18) | 0.378 | 0.486 | 0.95 | (±0.06) | 0.716 | 1.000 |
| DFFA: DNA fragmentation factor, 45 kD, alpha polypeptide | AA487452 | 1p36.3-p36.2 | **0.63**↓ | (±0.07) | 0.603 | 0.693 | 0.98 | (±0.27) | 0.756 | 1.000 |
| FCER1G: Fc fragment of IgE, high affinity I, receptor for; gamma polypeptide | H79353 | 1q23 | **0.66**↓ | (±0.10) | 0.202 | 0.310 | 1.24 | (±0.51) | 0.485 | 1.000 |
| DAF: decay accelerating factor for complement | R09561 | 1q32 | **0.66**↓ | (±0.13) | 0.136 | 0.269 | 0.98 | (±0.16) | 0.967 | 1.000 |
| CD8A: CD8 antigen, alpha polypeptide (p32) | AA443649 | 2p11.2 | **0.61**↓ | (±0.11) | 0.304 | 0.412 | 1.13 | (±0.41) | 0.843 | 1.000 |
| IL18R1: interleukin 18 receptor 1 | AA482637 | 2q12 | **0.676**↓ | (±0.25) | 0.387 | 0.495 | 1.12 | (±0.34) | 0.664 | 1.000 |
| NCF1: neutrophil cytosolic factor 1 (47kD, chronic granulomatous disease, autosomal 1) | AA489666 | 7q11.23 | **0.67**↓ | (±0.12) | 0.540 | 0.637 | 1.02 | (±0.25) | 0.858 | 1.000 |
| CLU: clusterin (complement lysis inhibitor, SP-40,40, sulfated glycoprotein 2, testosterone-repressed prostate message 2, apolipoprotein J) | AA130017 | 8p21-p12 | **0.65**↓ | (±0.07) | 0.673 | 0.751 | 1.01 | (±0.25) | 0.968 | 1.000 |
| C5: complement component 5 | N73030 | 9q32-q34 | **0.74**↓ | (±0.09) | 0.175 | 0.286 | **0.76**↓ | (±0.35) | 0.206 | 1.000 |
| PRDX5: peroxiredoxin 5 | N91311 | 11q13 | **0.59**↓ | (±0.20) | 0.283 | 0.389 | 1.14 | (±0.89) | 0.651 | 1.000 |
| PTPRCAP: protein tyrosine phosphatase, receptor type, C-associated protein | AA481547 | 11q13.3 | **0.73**↓ | (±0.23) | 0.554 | 0.649 | 1.01 | (±0.16) | 0.968 | 1.000 |
| CRADD: CASP2 and RIPK1 domain containing adaptor with death domain | R37937 | 12q21.33-q23.1 | **0.66**↓ | (±0.10) | 0.320 | 0.427 | 0.94 | (±0.32) | 0.685 | 1.000 |
| LTA4H: leukotriene A4 hydrolase | AA465366 | 12q22 | **0.76**↓ | (±0.10) | 0.137 | 0.269 | 1.00 | (±0.10) | 0.988 | 1.000 |
| TIAF1: TGFB1-induced anti-apoptotic factor 1 | AA446222 | 17q11.1-q11.2 | **0.70**↓ | (±0.10) | 0.379 | 0.487 | 1.04 | (±0.38) | 0.859 | 1.000 |
| BECN1: beclin 1 (coiled-coil, myosin-like BCL2 interacting protein) | AA427367 | 17q21 | **0.64**↓ | (±0.05) | 0.164 | 0.279 | **0.83**↓ | (±0.19) | 0.324 | 1.000 |
| CSF2RB: colony stimulating factor 2 receptor, beta, low-affinity (granulocyte-macrophage) | AA279147 | 22q13.1 | **0.51**↓ | (±0.25) | 0.115 | 0.265 | 0.93 | (±0.41) | 0.870 | 1.000 |
| CYBB: cytochrome b-245, beta polypeptide (chronic granulomatous disease) | AA463492 | Xp21.1 | **0.69**↓ | (±0.15) | 0.514 | 0.612 | 0.99 | (±0.16) | 0.873 | 1.000 |
| IL3RA:interleukin 3 receptor, alpha (low affinity) | W44701 | Xp22.3 or Yp11.3 | **0.80**↓ | (±0.13) | 0.748 | 0.814 | 1.19 | (±0.29) | 0.771 | 1.000 |

↓

| H: Organelle organization and biogenesis  GO:0006996 |  |  |  | | | |  | | | |
| --- | --- | --- | --- | --- | --- | --- | --- | --- | --- | --- |
| Gene name | GenBank no | chromosomal location | TGFβ−treated vs. control | | | | IMR-treated vs. control | | | |
|  |  |  | ratio | SD | p-value | FDR | ratio | SD | p-value | FDR |
| COG2: component of oligomeric golgi complex 2 | AA504526 | 1q42.2 | **0.74**↓ | (±0.22) | 0.063 | 0.265 | 1.06 | (±0.13) | 0.654 | 1.000 |
| TMSB10: thymosin, beta 10 | AA486085 | 2p11.2 | **0.63**↓ | (±0.05) | 0.142 | 0.269 | 0.96 | (±0.26) | 0.656 | 1.000 |
| PEX13: peroxisome biogenesis factor 13 | R16849 | 2p14-p16 | **0.61**↓ | (±0.22) | 0.556 | 0.650 | 1.14 | (±0.34) | 0.989 | 1.000 |
| AAMP: angio-associated, migratory cell protein | AA452988 | 2q36.1 | **0.58**↓ | (±0.09) | 0.047 | 0.265 | 1.11 | (±0.56) | 0.923 | 1.000 |
| TUBA1: tubulin, alpha 1 (testis specific) | AA180912 | 2q36.2 | **0.57**↓ | (±0.16) | 0.157 | 0.275 | 0.88 | (±0.23) | 0.435 | 1.000 |
| MYO6: myosin VI | AA028987 | 6q13 | **0.57**↓ | (±0.18) | 0.266 | 0.371 | 1.03 | (±0.17) | 0.947 | 1.000 |
| WAVE1: WAS protein family, member 1 | N59851 | 6q21-q22 | **0.64**↓ | (±0.11) | 0.457 | 0.562 | 1.00 | (±0.43) | 0.819 | 1.000 |
| COL5A1: collagen, type V, alpha 1 | R75635 | 9q35 | **0.64**↓ | (±0.04) | 0.618 | 0.704 | **0.78**↓ | (±0.28) | 0.783 | 1.000 |
| NRAP: nebulin-related anchoring protein | N48103 | 10q24-q26 | **0.74**↓ | (±0.11) | 0.700 | 0.774 | 1.04 | (±0.10) | 0.961 | 1.000 |
| CKAP5:cytoskeleton associated protein 5 | AA598942 | 11p11.2 | **0.62**↓ | (±0.21) | 0.586 | 0.678 | 1.31 | (±0.39) | 0.710 | 1.000 |
| CD151: CD151 antigen | AA456183 | 11p15.5 | **0.59**↓ | (±0.08) | 0.263 | 0.369 | 0.92 | (±0.30) | 0.575 | 1.000 |
| LUM: lumican | AA453712 | 12q21.3-q22 | **0.63**↓ | (±0.13) | 0.137 | 0.269 | 1.11 | (±0.40) | 0.778 | 1.000 |
| PCDH20: Protocadherin 20 | AA040043 | 13q21 | **0.54**↓ | (±0.16) | 0.419 | 0.527 | 0.99 | (±0.29) | 0.809 | 1.000 |
| LAMP1: lysosomal-associated membrane protein 1 | H29077 | 13q34 | **0.75**↓ | (±0.21) | 0.504 | 0.637 | 1.06 | (±0.28) | 0.958 | 1.000 |
| GFAP: Glial fibrillary acidic protein | AA069414 | 17q21 | **0.69**↓ | (±0.14) | 0.161 | 0.277 | 0.99 | (±0.17) | 0.949 | 1.000 |
| PNUTL2: peanut-like 2 (Drosophila), | T64878 | 17q22-q23 | **0.81**↓ | (±0.15) | 0.408 | 0.514 | **1.15**↑ | (±0.08) | 0.505 | 1.000 |
| DSC2: Desmocollin 2 | AA074677 | 18q11.2 | **0.55**↓ | (±0.27) | 0.153 | 0.274 | 1.06 | (±0.73) | 0.597 | 1.000 |
| CNN2: calponin 2 | AA284568 | 19p13.3 | **0.69**↓ | (±0.10) | 0.197 | 0.305 | 0.90 | (±0.15) | 0.604 | 1.000 |
| CKAP1: cytoskeleton associated protein 1 | AA504554 | 19q13.11-q13.12 | **0.72**↓ | (±0.14) | 0.239 | 0.344 | 1.18 | (±0.29) | 0.555 | 1.000 |
| JAM2: junctional adhesion molecule 2 | R68464 | 21q21.2 | **0.87**↓ | (±0.24) | 0.730 | 0.800 | 1.19 | (±0.42) | 0.513 | 1.000 |
| COL18A1: collagen, type XVIII, alpha 1 | W07798 | 21q22.3 | **0.57**↓ | (±0.17) | 0.164 | 0.279 | **0.84**↓ | (±0.22) | 0.411 | 1.000 |
| BGN: biglycan | N51018 | Xq28 | **2.95**↑ | (±1.56) | 0.623 | 0.708 | 1.15 | (±0.73) | 0.575 | 1.000 |

| I) Transport  GO:0006810 |  |  |  | | | |  | | | |
| --- | --- | --- | --- | --- | --- | --- | --- | --- | --- | --- |
| Gene name | GenBank no | chromosomal location | TGFβ−treated vs. control | | | | IMR-treated vs. control | | | |
|  |  |  | ratio | SD | p-value | FDR | ratio | SD | p-value | FDR |
| SLC35A3: solute carrier family 35 (UDP-N-acetylglucosamine (UDP-GlcNAc) transporter), member A3 | AA034501 | 1p21 | **0.80**↓ | (±0.18) | 0.117 | 0.265 | **1.30**↑ | (±0.39) | 0.138 | 1.000 |
| ATP6V0B: ATPase, H+ transporting, lysosomal 21kD, V0 subunit c" | AA480826 | 1p32.3 | **0.69**↓ | (±0.05) | 0.213 | 0.320 | **0.84**↓ | (±0.26) | 0.244 | 1.000 |
| RAB1A: RAB1A, member RAS oncogene family | N69689 | 2p14 | **0.52**↓ | (±0.22) | 0.371 | 0.479 | 0.89 | (±0.27) | 0.612 | 1.000 |
| ATP6V1C2: ATPase, H+ transporting, lysosomal 42kDa, V1 subunit C isoform 2 | R02609 | 2p25 | **0.64**↓ | (±0.01) | 0.225 | 0.331 | 1.04 | (±0.66) | 0.936 | 1.000 |
| BZAP45: basic leucine-zipper protein BZAP45 | AA463591 | 2q33 | **0.46**↓ | (±0.25) | 0.151 | 0.273 | **0.73**↓ | (±0.30) | 0.259 | 1.000 |
| CACNA1D: Calcium channel, voltage-dependent, L type, alpha 1D subunit | H29256 | 3p14.3 | **0.60**↓ | (±0.23) | 0.385 | 0.492 | 1.12 | (±0.56) | 0.855 | 1.000 |
| SEC22A: Sec22 homolog | W47156 | 3q21.1 | **0.62**↓ | (±0.15) | 0.261 | 0.367 | 1.08 | (±0.56) | 0.799 | 1.000 |
| ATP1B3: ATPase, Na+/K+ transporting, beta 3 polypeptide | AA489275 | 3q22-q23 | **0.66**↓ | (±0.15) | 0.294 | 0.401 | 1.06 | (±0.15) | 0.831 | 1.000 |
| TFRC: transferrin receptor (p90, CD71) | AA488721 | 3q29 | **0.73**↓ | (±0.10) | 0.329 | 0.435 | 1.01 | (±0.31) | 0.866 | 1.000 |
| P115: vesicle docking protein p115 | AA504342 | 4q21.1 | 0.83 | (±0.30) | 0.448 | 0.553 | **1.30**↑ | (±0.21) | 0.041 | 1.000 |
| FRDA: Friedreich ataxia | AA253413 | 9q13-q21.1 | **0.54**↓ | (±0.15) | 0.158 | 0.276 | 0.97 | (±0.29) | 0.712 | 1.000 |
| FOLR1: folate receptor 1 (adult) | R24635 | 11q13.3-q14.1 | 0.86 | (±0.45 | 0.384 | 0.492 | **0.67**↓ | (±0.37) | 0.193 | 1.000 |
| SLC6A12: solute carrier family 6 (neurotransmitter transporter, betaine/GABA), member 12 | N49856 | 12p13 | **0.82**↓ | (±0.20) | 0.553 | 0.648 | 1.15 | (±0.49) | 0.795 | 1.000 |
| VPS4A: Vacuolar protein sorting 4A (yeast) | W79674 | 16q22 | **0.51**↓ | ↓ (±0.36) | 0.130 | 0.268 | 0.85 | (±0.57) | 0.332 | 1.000 |
| SLC13A5: Solute carrier family 13 (sodium-dependent citrate transporter), member 5 | N80622 | 17p13 | **4.54**↑ | (±2.57) | 0.208 | 0.315 | 0.88 | (±0.70) | 0.534 | 1.000 |
| ATP6V0A1: ATPase, H+ transporting, lysosomal V0 subunit a isoform 1 | AA427472 | 17q21 | **0.65**↓  **0.62**↓ | (±0.08)  (±0.02) | 0.588  0.256 | 0.680  0.361 | 0.92  0.90 | (±0.27)  (±0.22) | 0.971  0.738 | 1.000  1.000 |
| PCTP: phosphatidylcholine transfer protein | AA030013 | 17q21-q24 | **0.81**↓ | (±0.31) | 0.570 | 0.663 | 1.13 | (±0.25) | 0.815 | 1.000 |
| RAB37: RAB37, member RAS oncogene family | R06033 | 17q25 | **0.67**↓ | (±0.01) | 0.607 | 0.695 | 1.33 | (±0.85) | 0.916 | 1.000 |
| PRG1: Proteoglycan 1, secretory granule | AA278759 | 19q13.2 | **0.80**↓ | (±0.14) | 0.797 | 0.852 | 1.25 | (±0.34) | 0.611 | 1.000 |
| SLC16A2: solute carrier family 16 (monocarboxylic acid transporters), member 2 | AA425612 | Xq13.2 | **0.64**↓ | (±0.07) | 0.034 | 0.265 | 0.90 | (±0.15) | 0.613 | 1.000 |
| ATP6IP1: ATPase, H+ transporting, lysosomal interacting protein 1 | AA488715 | Xq28 | **0.44**↓ | (±0.32) | 0.205 | 0.312 | **0.84**↓ | (±0.48) | 0.391 | 1.000 |

| J) Biological process unknown  GO:0000004 |  |  |  | | | |  | | | |
| --- | --- | --- | --- | --- | --- | --- | --- | --- | --- | --- |
| Gene name | GenBank no | chromosomal location | TGFβ−  treated vs. control | | | | IMR-treated vs. control | | | |
|  |  |  | ratio | SD | p-value | FDR | ratio | SD | p-value | FDR |
| LOC440582: similar to Peptidyl-prolyl cis-trans isomerase E (PPIase E) (Rotamase E) (Cyclophilin E) (Cyclophilin 33) | W17246 | 1p34.3 | **0.79**↓ | (±0.20) | 0.290 | 0.397 | 1.11 | (±0.14) | 0.293 | 1.000 |
| ZNF258: zinc finger, MYM-type 6 | H65044 | 1p34 | **3.64**↑ | (±1.89) | 0.305 | 0.413 | 0.77 | (±0.55) | 0.465 | 1.000 |
| GNL2: guanine nucleotide binding protein-like 2 (nucleolar) | AA446682 | 1p34.3 | **0.63**↓ | (±0.03) | 0.483 | 0.587 | 1.17 | (±0.39) | 0.687 | 1.000 |
| MANEAL: mannosidase, endo-alpha-like | W24055 | 1p34.3 | **0.61**↓ | (±0.14) | 0.296 | 0.403 | 1.28 | (±1.10) | 0.804 | 1.000 |
| RP4-622L5: hypothetical protein RP4-622L5 | T85191 | 1p36.11-p34.2 | **0.69**↓ | (±0.13) | 0.066 | 0.265 | 1.11 | (±0.37) | 0.673 | 1.000 |
| INTS7: integrator complex subunit 7 | N80458 | 1p36.13-q42.3 | **0.59**↓ | (±0.21) | 0.456 | 0.560 | 0.93 | (±0.31) | 0.659 | 1.000 |
| CHI3L1: chitinase 3-like 1 (cartilage glycoprotein-39) | AA434115 | 1q32.1 | **0.75**↓ | (±0.25) | 0.221 | 0.328 | 0.86 | (±0.06) | 0.510 | 1.000 |
| LRCC39: leucine rich repeat containing 39 | W06875 | 1pter-q31.3 | 1.08 | (±1.16) | 0.790 | 0.847 | **1.45**↑ | (±0.46) | 0.432 | 1.000 |
| FLJ32312: hypothetical protein FLJ32312 | W23571 | 2p16.1 | **0.62**↓ | (±0.19) | 0.677 | 0.754 | 0.94 | (±0.22) | 0.785 | 1.000 |
| EST | R24356 | 2p21 | **0.65**↓ | (±0.33) | 0.401 | 0.508 | 0.89 | (±0.50) | 0.541 | 1.000 |
| EST | R63530 | 2p23 | **0.69**↓ | (±0.07) | 0.226 | 0.332 | 0.84 | (±0.25) | 0.532 | 1.000 |
| GLI2: GLI-Kruppel family member GLI2 | H70114 | 2q14 | **4.15**↑ | (±1.93) | 0.235 | 0.340 | 0.87 | (±0.56) | 0.563 | 1.000 |
| RPL31: Ribosomal protein L31 | W15277 | 2q14 | **0.54**↓ | (±0.26) | 0.085 | 0.265 | 0.97 | (±0.39) | 0.651 | 1.000 |
| PSCDBP: pleckstrin homology, Sec7 and coiled/coil domains, binding protein | AA490903 | 2q11.2 | **0.50**↓ | (±0.36) | 0.402 | 0.509 | 0.93 | (±0.56) | 0.567 | 1.000 |
| FLJ37953 | W04206 | 2q33 | **0.73**↓ | (±0.05) | 0.347 | 0.454 | 0.99 | (±0.42) | 0.737 | 1.000 |
| PREI3: preimplantation protein 3 | W76032 | 2q33.1 | **0.72**↓ | (±0.16) | 0.624 | 0.708 | 1.11 | (±0.11) | 0.837 | 1.000 |
| EST | T99671 | 2q36 | **0.66**↓ | (±0.18) | 0.610 | 0.698 | 0.99 | (±0.30) | 0.770 | 1.000 |
| CCDC48:coiled-coil domain containing 48 | N77990 | 3q21 | **0.71**↓ | (±0.10) | 0.394 | 0.501 | 1.01 | (±0.26) | 0.840 | 1.000 |
| FLJ10707 | N91677 | 3p25.3 | **0.83**↓ | (±0.17) | 0.379 | 0.486 | 1.21 | (±0.13) | 0.142 | 1.000 |
| SELT: selenoprotein T | R78516 | 3q25.1 | **0.80**↓ | (±0.17) | 0.058 | 0.265 | 0.96 | (±0.18) | 0.850 | 1.000 |
| MGC2198 | H81199 | 3q27-q28 | **0.57**↓ | (±0.25) | 0.258 | 0.364 | 1.21 | (±0.97) | 0.727 | 1.000 |
| IMP-2: IGF-II mRNA-binding protein 2 | N71442 | 3q27.2 | **0.63**↓ | (±0.25) | 0.546 | 0.642 | 1.17 | (±0.52) | 0.995 | 1.000 |
| EST | T95462 | 3q28 | **0.66**↓ | (±0.13) | 0.136 | 0.269 | 1.01 | (±0.17) | 0.959 | 1.000 |
| Hypothetical LOC389188 | R28424 | 3q29 | **0.61**↓ | (±0.21) | 0.082 | 0.265 | 0.85 | (±0.35) | 0.479 | 1.000 |
| EST | R00591 | 4p16.1 | **3.20**↑ | (±1.50) | 0.445 | 0.550 | **0.78**↓ | (±0.48) | 0.455 | 1.000 |
| RIPX: rap2 interacting protein x | R74171 | 4q21.1 | **0.62**↓ | (±0.20) | 0.175 | 0.287 | **0.68**↓ | (±0.25) | 0.251 | 1.000 |
| EST | H94236 | 4q35 | **3.51**↑ | (±1.59) | 0.383 | 0.491 | 0.95 | (±0.66) | 0.493 | 1.000 |
| EST | R74480 | 5 | **0.65**↓ | (±0.09) | 0.148 | 0.272 | 0.93 | (±0.55) | 0.458 | 1.000 |
| CCL28: chemokine (C-C motif) ligand 28 | R38459 | 5p12 | **0.63**↓ | (±0.13) | 0.288 | 0.395 | 1.06 | (±0.41) | 0.905 | 1.000 |
| FCHO2: FCH domain only 2 | H93842 | 5q13 | 1.03 | (±0.54) | 0.859 | 0.900 | **1.48**↑ | (±0.44) | 0.277 | 1.000 |
| KIAA0372 | AA233339 | 5q14.3 | **0.55**↓ | (±0.20) | 0.503 | 0.602 | 0.88 | (±0.42) | 0.927 | 1.000 |
| C5orf13: chromosome 5 open reading frame 13 | N91952 | 5q22 | **0.58**↓ | (±0.08) | 0.153 | 0.274 | 1.25 | (±0.85) | 0.841 | 1.000 |
| EST: ALU8_HUMAN Alu subfamily SX | R36624 | 5q22-q23 | **0.68**↓ | (±0.22) | 0.234 | 0.338 | 1.21 | (±0.41) | 0.520 | 1.000 |
| Hypothetical gene supported by AK126569 | N92035 | 5q23 | **0.52**↓ | (±0.20) | 0.161 | 0.277 | 0.94 | (±0.31) | 0.577 | 1.000 |
| ABLIM3: Actin binding LIM protein family, member 3 | N90968 | 5q32 | **3.75**↑ | (±2.48) | 0.595 | 0.685 | 0.90 | (±0.86) | 0.444 | 1.000 |
| SMG1: PI-3-kinase-related kinase SMG-1 | W32907 | 6p12.3 | **0.70**↓ | (±0.22) | 0.207 | 0.315 | 1.36 | (±0.55) | 0.259 | 1.000 |
| KIAA0082 protein | AA504534 | 6p21.1 | **0.66**↓ | (±0.13) | 0.123 | 0.265 | 1.03 | (±0.24) | 0.950 | 1.000 |
| ATP13A5: ATPase type 13A5 | H16573 | 6p21.1 | **0.76**↓ | (±0.15) | 0.605 | 0.694 | 1.30 | (±0.66) | 0.656 | 1.000 |
| PSPHL: phosphoserine phosphatase-like | W05628 | 7q11.2 | **0.76**↓ | (±0.12) | 0.263 | 0.341 | 1.14 | (±0.18) | 0.583 | 1.000 |
| FLJ32731: transmembrane protein 76 | R71531 | 8p11.1 | **0.83**↓ | (±0.20) | 0.899 | 0.923 | 1.13 | (±0.94) | 0.769 | 1.000 |
| cDNA DKFZp667D095 | R26163 | 8p22 | **0.61**↓ | (±0.32) | 0.118 | 0.265 | 1.10 | (±0.59) | 0.857 | 1.000 |
| RRS1: RRS1 ribosome biogenesis regulator homolog (S. cerevisiae) | AA100612 | 8q13.1 | **4.39**↑ | (±2.61) | 0.311 | 0.418 | 2.92 | (±2.73) | 0.494 | 1.000 |
| KIAA0146: KIAA0146 protein | AA401448 | 8q11.21 | **0.64**↓ | (±0.12) | 0.407 | 0.514 | 1.05 | (±0.42) | 0.782 | 1.000 |
| LOC157567: ankyrin repeat domain 46 | AA234889 | 8q22 | **0.65**↓ | (±0.37) | 0.487 | 0.589 | 1.00 | (±0.41) | 0.696 | 1.000 |
| DPYS: hypothetical protein PRO2949 | N73761 | 8q22 | **3.29**↑ | (±1.73) | 0.513 | 0.611 | 0.85 | (±0.54) | 0.566 | 1.000 |
| C9orf41: chromosome 9 open reading frame 41 | H50724 | 9q21.13 | **0.80**↓ | (±0.03 | 0.735 | 0.804 | 1.01 | (±0.25) | 0.954 | 1.000 |
| TMOD1: tropomodulin 1 | AA410680 | 9q22.3 | **0.59**↓ | (±0.23) | 0.194 | 0.303 | 0.90 | (±0.42) | 0.494 | 1.000 |
| RAPGEF1: rap guanine nucleotide exchange factor (GEF) 1 | R91271 | 9q34 | **0.67**↓ | (±0.05) | 0.111 | 0.265 | 0.93 | (±0.10) | 0.747 | 1.000 |
| SURF5 | AA459247 | 9q34.2 | **0.61**↓ | (±0.18) | 0.195 | 0.304 | 1.04 | (±0.60) | 0.663 | 1.000 |
| C10orf7: chromosome 10 open reading frame 7 | AA448289 | 10p13 | **0.77**↓ | (±0.17) | 0.797 | 0.852 | 1.20 | (±0.22) | 0.896 | 1.000 |
| TMEM23: transmembrane protein 23 | R23222 | 10q11.2 | **0.53**↓ | (±0.19) | 0.245 | 0.350 | 1.00 | (±0.70) | 0.544 | 1.000 |
| UVRAG: UV radiation resistance associated gene | AA490771 | 11q13.5 | **0.77**↓ | (±0.17) | 0.089 | 0.265 | 0.99 | (±0.10) | 0.843 | 1.000 |
| LOC399979: similar to Sorting nexin 19 | AA040424 | 11q24.3 | **3.57**↑ | (±1.92) | 0.434 | 0.540 | 0.94 | (±0.86) | 0.524 | 1.000 |
| ROBO3: roundabout, axon guidance receptor, homolog 3 (Drosophila) | N80769 | 11q24.2 | **0.65**↓ | (±0.20) | 0.485 | 0.588 | 1.20 | (±0.45) | 0.846 | 1.000 |
| ATF7IP: activating transcription factor 7 interacting protein | R31512 | 12p13.1 | **0.67**↓ | (±0.14) | 0.434 | 0.540 | 1.24 | (±0.61) | 0.990 | 1.000 |
| JARID1A: Jumonji, AT rich interactive domain 1A (RBBP2-like) | W74352 | 12p13 | **0.61**↓ | (±0.15) | 0.029 | 0.265 | 1.13 | (±0.80) | 0.957 | 1.000 |
| KIAA1467 protein | W33011 | 12p13.2 | **0.59**↓ | (±0.13) | 0.049 | 0.265 | 1.03 | (±0.37) | 0.877 | 1.000 |
| D12S2489E | AA397819 | 12p13.2-p12.3 | **0.66**↓ | (±0.06) | 0.011 | 0.227 | 0.92 | (±0.19) | 0.678 | 1.000 |
| EST | H63361 | 12q24.31 | **3.50**↑ | (±2.03) | 0.271 | 0.376 | **0.66**↓ | (±0.26) | 0.520 | 1.000 |
| METTL1: methyltransferase-like 1 | AA422058 | 12q13 | **0.76**↓ | (±0.26) | 0.150 | 0.273 | 1.04 | (±0.04) | 0.780 | 1.000 |
| PWP1: nuclear phosphoprotein similar to S. cerevisiae PWP1 | AA485992 | 12q23.3 | 0.82 | (±0.27) | 0.852 | 0.895 | **1.30**↑ | (±0.10) | 0.592 | 1.000 |
| LHFP: lipoma HMGIC fusion partner | N58145 | 13q12 | **3.49**↑ | (±1.61) | 0.291 | 0.398 | 0.89 | (±0.54) | 0.593 | 1.000 |
| LOC440135 | R84242 | 13q14.13 | **0.52**↓ | (±0.29) | 0.231 | 0.336 | 1.77 | (±2.21) | 0.717 | 1.000 |
| FLJ22624: FLJ22624 protein | R23924 | 13q21.33 | **0.61**↓ | (±0.13) | 0.420 | 0.527 | 1.35 | (±1.07) | 0.878 | 1.000 |
| COMMD6: COMM domain containing 6 | AA029889 | 13q22 | **0.58**↓ | (±0.34) | 0.126 | 0.267 | 1.13 | (±0.76) | 0.982 | 1.000 |
| SAMD4A:sterile alpha motif domain containing 4A | R96525 | 14q22 | **3.15**↑ | (±1.77) | 0.431 | 0.537 | 0.86 | (±0.49) | 0.499 | 1.000 |
| FLJ38426: family with sequence similarity 98, member B | W47254 | 15q14 | 0.84 | (±0.15) | 0.723 | 0.794 | **1.25**↑ | (±0.09) | 0.452 | 1.000 |
| DKFZP564G2022: transmembrane protein 87A | N63753 | 15q15 | **0.62**↓ | (±0.22) | 0.745 | 0.813 | 1.26 | (±0.46) | 0.911 | 1.000 |
| FLJ31407 fis, clone NT2NE2000137 | H58949 | 15q21 | **3.84**↑ | (±2.11) | 0.402 | 0.509 | 0.90 | (±0.93) | 0.460 | 1.000 |
| ATXN2L:ataxin 2-like | AA029963 | 16p11.1 | **0.71**↓ | (±0.19) | 0.405 | 0.512 | 1.02 | (±0.04) | 0.963 | 1.000 |
| ZNF629: zinc finger protein 629 | AA128587 | 16p11.1 | **0.55**↓ | (±0.15) | 0.157 | 0.275 | 0.93 | (±0.33) | 0.533 | 1.000 |
| MGC5178 | N55087 | 16p12 | **0.53**↓ | (±0.22) | 0.265 | 0.370 | 1.09 | (±0.60) | 0.848 | 1.000 |
| FTS: fused toes homolog (mouse) | W52803 | 16q12.2 | **0.66**↓ | (±0.07) | 0.328 | 0.433 | 0.98 | (±0.17) | 0.827 | 1.000 |
| LOC124446: hypothetical protein BC017488 | W93317 | 16q13 | **0.60**↓ | (±0.05) | 0.006 | 0.192 | **0.80**↓ | (±0.24) | 0.345 | 1.000 |
| cDNA FLJ32121 fis, clone PEBLM1000083 | T69477 | 16q22 | **0.57**↓ | (±0.18) | 0.124 | 0.265 | 0.85 | (±0.25) | 0.698 | 1.000 |
| ACD: adrenocortical dysplasia homolog (mouse) | H79234 | 16q22.3 | **0.69**↓ | (±0.05) | 0.573 | 0.665 | 1.27 | (±0.54) | 0.842 | 1.000 |
| NDEL1: nudE nuclear distribution gene E homolog like 1 (A. nidulans) | R94775 | 17p13.1 | **0.79**↓ | (±0.23) | 0.243 | 0.347 | 1.05 | (±0.17) | 0.841 | 1.000 |
| UNC119: unc-119 homolog (C. elegans) | AA457199 | 17q11.2 | **0.75**↓ | (±0.10) | 0.383 | 0.491 | 0.94 | (±0.18) | 0.650 | 1.000 |
| C18ORF1: chromosome 18 open reading frame 1 | AA489736 | 18p11.2 | **0.58**↓ | (±0.07) | 0.131 | 0.268 | 0.94 | (±0.33) | 0.586 | 1.000 |
| SFRS14: splicing factor, arginine/serine-rich 14 | AA485539 | 19p12 | **0.70**↓ | (±0.17) | 0.201 | 0.310 | 1.88 | (±1.38) | 0.995 | 1.000 |
| DKFZP434D1335 | H25229 | 19q13.12 | **3.40**↑ | (±1.79) | 0.520 | 0.618 | 0.84 | (±0.51) | 0.510 | 1.000 |
| FLJ10922: hypothetical protein FLJ10922 | H83094 | 19q13.33 | **0.46**↓ | (±0.23) | 0.334 | 0.440 | 0.96 | (±0.38) | 0.688 | 1.000 |
| PTOV1 prostate tumour over expressed gene 1 | AA486332 | 19q13.33 | **0.63**↓ | (±0.08) | 0.090 | 0.265 | **0.84**↓ | (±0.17) | 0.618 | 1.000 |
| C20orf155: chromosome 20 open reading frame 155 | R06311 | 20p13 | **3.45**↑ | (±2.65) | 0.704 | 0.777 | 1.27 | (±0.86) | 0.557 | 1.000 |
| C20orf129: chromosome 20 open reading frame 129 | R96941 | 20q11.22-q12 | **0.55**↓ | (±0.27) | 0.073 | 0.265 | 1.17 | (±0.66) | 0.755 | 1.000 |
| TPD52L2: tumor protein D52-like 2 | R06309 | 20q13.2-q13.3 | **0.52**↓ | (±0.20) | 0.137 | 0.265 | 1.65 | (±1.49) | 0.582 | 1.000 |
| C21orf4: chromosome 21 open reading frame 4 | N90335 | 21q22.11 | **0.62**↓ | (±0.19) | 0.553 | 0.648 | 1.23 | (±0.68) | 0.960 | 1.000 |
| PNPLA4: patatin-like phospholipase domain containing 4 | AA449678 | Xp22.3 | **0.58**↓ | (±0.09) | 0.387 | 0.495 | 0.94 | (±0.33) | 0.634 | 1.000 |
| HDHD1A: haloacid dehalogenase-like hydrolase domain containing 1A | AA278240 | Xp22.32 | **0.63**↓ | (±0.34) | 0.224 | 0.330 | 0.85 | (±0.53) | 0.387 | 1.000 |
| CXorf45: chromosome X open reading frame 45 | AA281346 | Xq23 | **0.57**↓ | (±0.20) | 0.046 | 0.265 | 1.05 | (±0.61) | 0.889 | 1.000 |
| LOC286467: hypothetical protein LOC286467 | R95805 | Xq26.1 | **2.85**↑ | (±1.02) | 0.293 | 0.400 | **0.85**↓ | (±0.42) | 0.530 | 1.000 |
| CXorf12: chromosome X open reading frame 12 | AA455272 | Xq28 | **0.74**↓ | (±0.10) | 0.395 | 0.502 | 0.85 | (±0.18) | 0.710 | 1.000 |
| EST | N77652 |  | **0.70**↓ | (±0.14) | 0.608 | 0.696 | 1.07 | (±0.12) | 0.908 | 1.000 |
| EST | R25153 |  | **0.61**↓ | (±0.04) | 0.058 | 0.265 | 1.06 | (±0.69) | 0.900 | 1.000 |

Genes are sorted by functional classification. A: Cellular metabolism (GO:0044237), Metabolism (other than energy metabolism), B: Generation of precursor metabolites and energy (GO:0006091), C: Nucleic acid binding (GO:0003676), D: Transcription regulator activity (GO:0030528), E: Signal transducer activity (GO:0004871), Signal transduction (GO:0007165) and Cell communication (GO:0007154), F: Cellular macromolecule metabolism (GO:0044260),G: Inflammatory response (GO:0006954), H: Organelle organization and biogenesis (GO:0006996I), Transport (GO:0006810), J) Biological process unknown (GO:0000004), Upward arrow denotes up-regulated mRNA expression, downward arrow down-regulated mRNA expression, bold indicates genes that have significant alteration in gene expression. Ratio is mean value calculated from three separate microarray experiments; SD is the standard deviation between separate experiments. Statistic probability, p-value, was calculated by using t-test. FDR is the calculated Benjamin-Hochberg false discovery rate.
