# Supplementary figures and images for: Gene expression in TGFbeta-induced epithelial cell differentiation in a three-dimensional intestinal epithelial cell differentiation model
Source: BMC Genomics. 2006 Oct 31;7:279. doi: 10.1186/1471-2164-7-279 (PMC1635984; doi:10.1186/1471-2164-7-279)

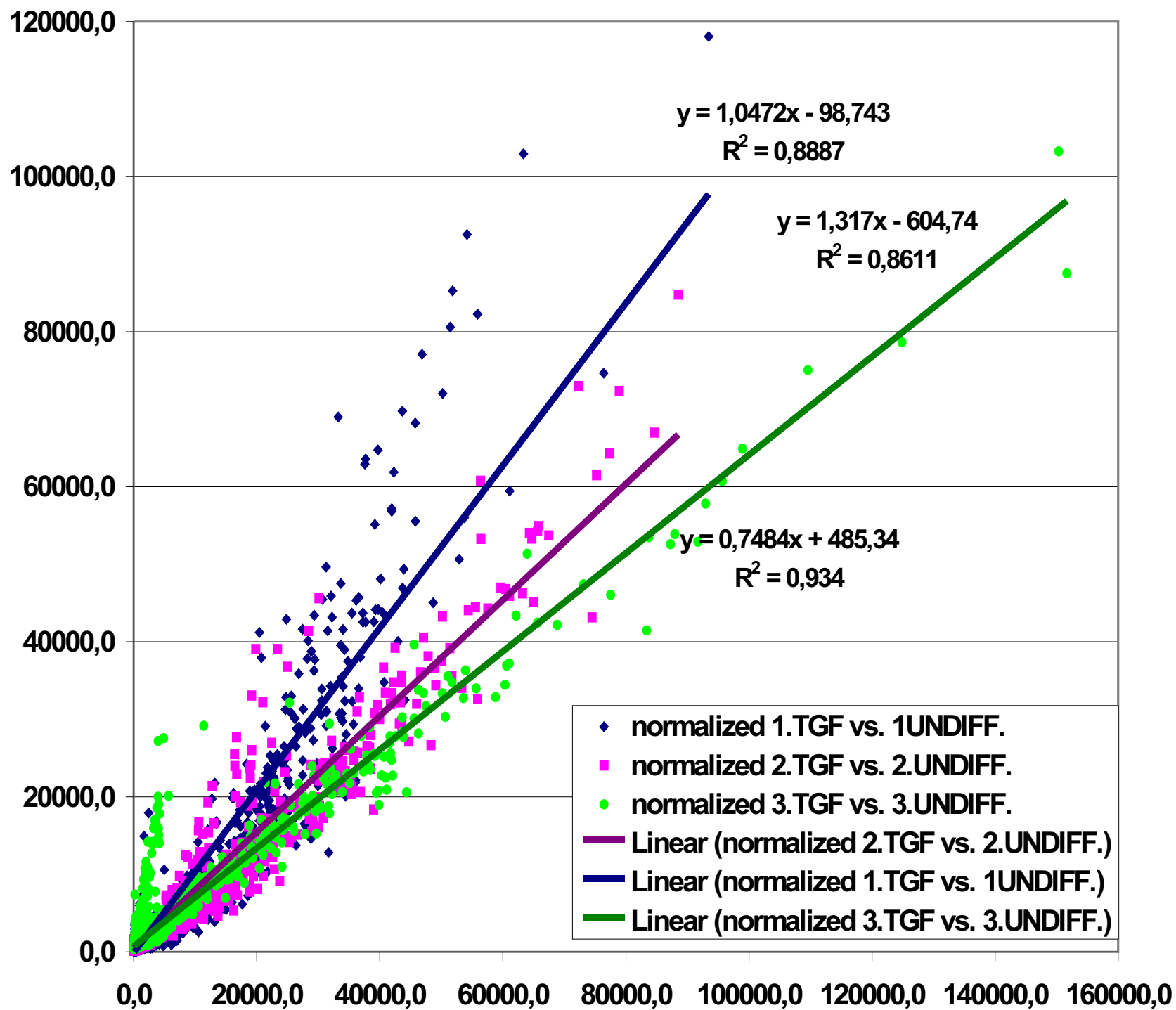

Supplement: Additional file 1 — The scatter plot for the normalized data: TGFβ-differentiated compared to undifferentiated T84 cells grown solely in collagen I gel. [file 1471-2164-7-279-S1.pdf]

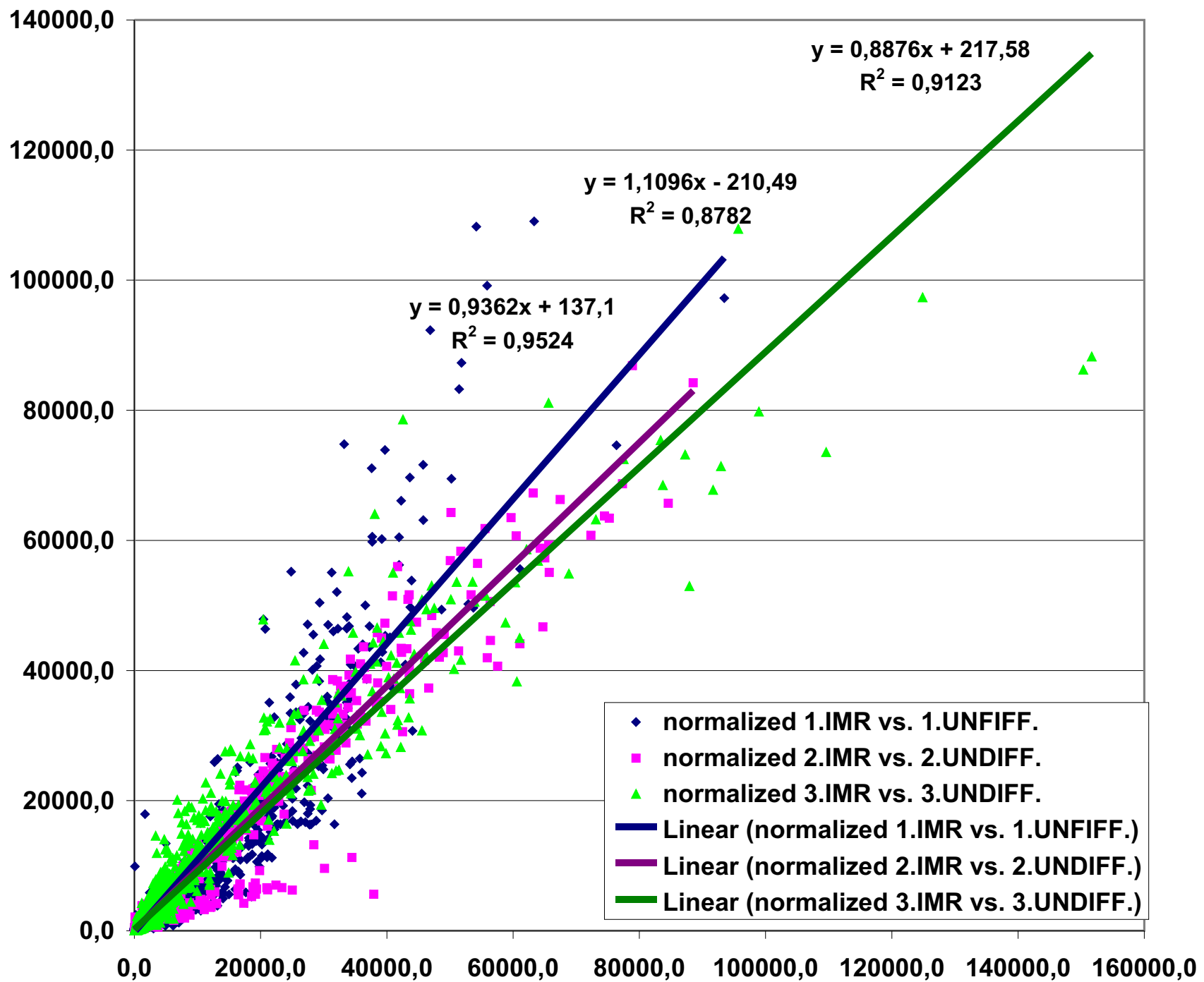

Supplement: Additional file 2 — The scatter plot for the normalized data:T84 cells differentiated by soluble factors secreted by mesenchymal cells compared to undifferentiated T84 cells grown solely in collagen I gel. [file 1471-2164-7-279-S2.pdf]

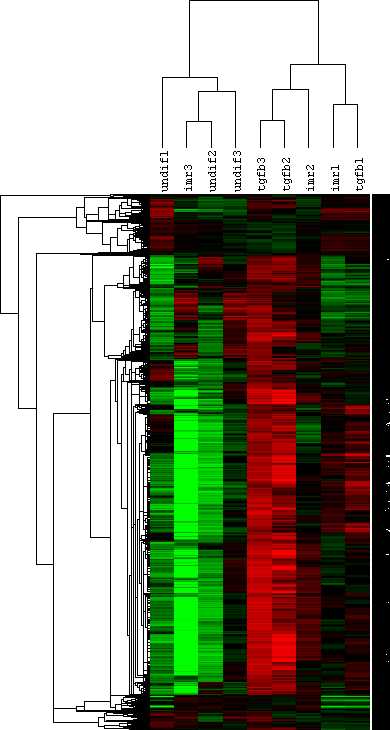

Supplement: Additional file 3 — Cluster analysis for unfiltered data. Clustering of genes was done with software programs Cluster and Treeview. [file 1471-2164-7-279-S3.png]

Color Key

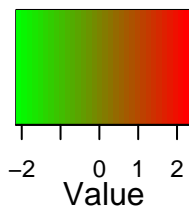

## Differentially expressed genes

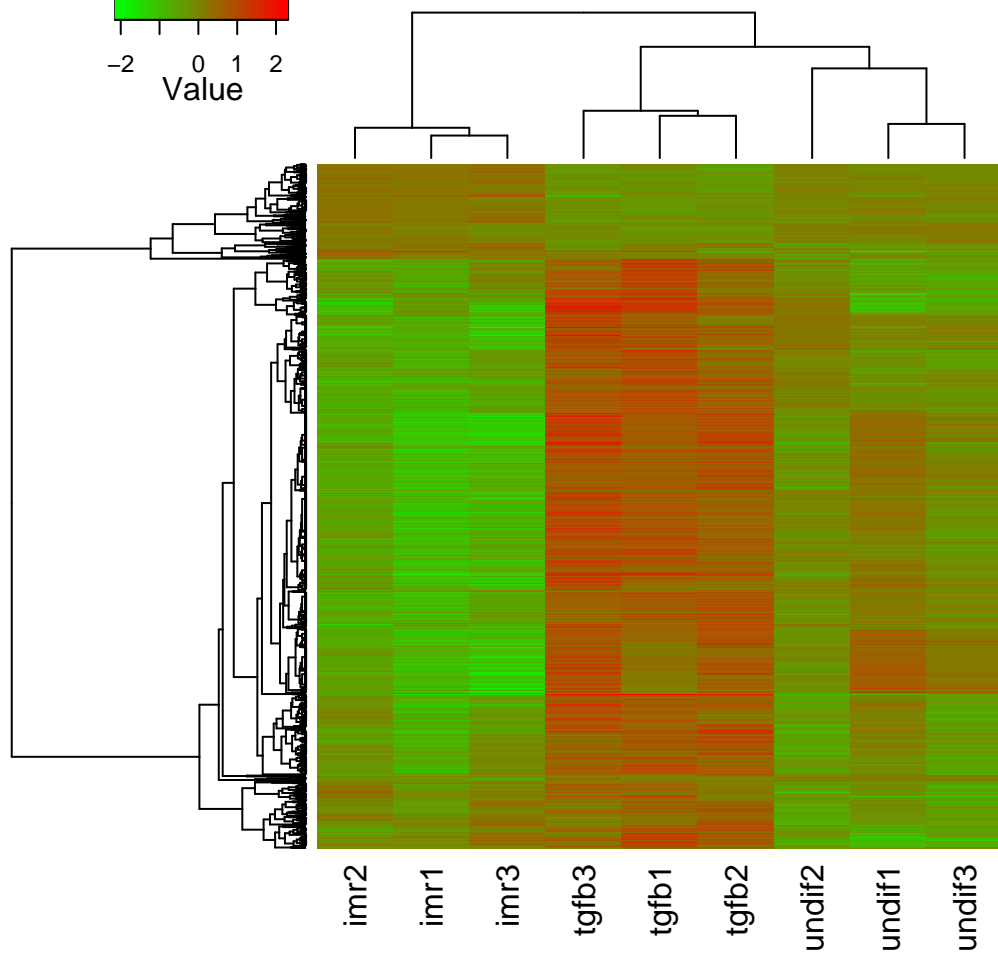

Supplement: Additional file 4 — Cluster analysis, heatmap, for the data first normalized by the sum method and further normalized using a linear mixed model. The Clustering of genes was done with software programs Cluster and Treeview. [file 1471-2164-7-279-S4.pdf]
